# Adherens junction proteins on the move—From the membrane to the nucleus in intestinal diseases

**DOI:** 10.3389/fcell.2022.998373

**Published:** 2022-10-05

**Authors:** Lindyann R. Lessey, Shaiya C. Robinson, Roopali Chaudhary, Juliet M. Daniel

**Affiliations:** Department of Biology, McMaster University, Hamilton, ON, Canada

**Keywords:** Adherens Junction, E-cadherin, catenins, Kaiso, Wnt signaling, inflammatory bowel disease, colon cancer

## Abstract

The function and structure of the mammalian epithelial cell layer is maintained by distinct intercellular adhesion complexes including *adherens* junctions (AJs), tight junctions, and desmosomes. The AJ is most integral for stabilizing cell-cell adhesion and conserving the structural integrity of epithelial tissues. AJs are comprised of the transmembrane protein E-cadherin and cytoplasmic catenin cofactors (α, β, γ, and p120-catenin). One organ where malfunction of AJ is a major contributor to disease states is the mammalian intestine. In the intestine, cell-cell adhesion complexes work synergistically to maintain structural integrity and homeostasis of the epithelium and prevent its malfunction. Consequently, when AJ integrity is compromised in the intestinal epithelium, the ensuing homeostatic disruption leads to diseases such as inflammatory bowel disease and colorectal carcinoma. In addition to their function at the plasma membrane, protein components of AJs also have nuclear functions and are thus implicated in regulating gene expression and intracellular signaling. Within the nucleus, AJ proteins have been shown to interact with transcription factors such as TCF/LEF and Kaiso (*ZBTB33*), which converge on the canonical Wnt signaling pathway. The multifaceted nature of AJ proteins highlights their complexity in modulating homeostasis and emphasizes the importance of their subcellular localization and expression in the mammalian intestine. In this review, we summarize the nuclear roles of AJ proteins in intestinal tissues; their interactions with transcription factors and how this leads to crosstalk with canonical Wnt signaling; and how nuclear AJ proteins are implicated in intestinal homeostasis and disease.

## Introduction

The gastrointestinal (GI) tract is a hollow continuous tube that begins at the mouth and ends at the anus. Digestion, waste elimination and nutrient absorption occurs over the folded epithelia of the small and large intestines (Michielan and D'Incà, 2015; Peterson and Artis, 2014). The epithelial layer is comprised of intestinal epithelial cells (IEC) that undergo rapid turnover and are replenished, on average, every 3–5 days (Rees et al., 2020).

**FIGURE 1:**
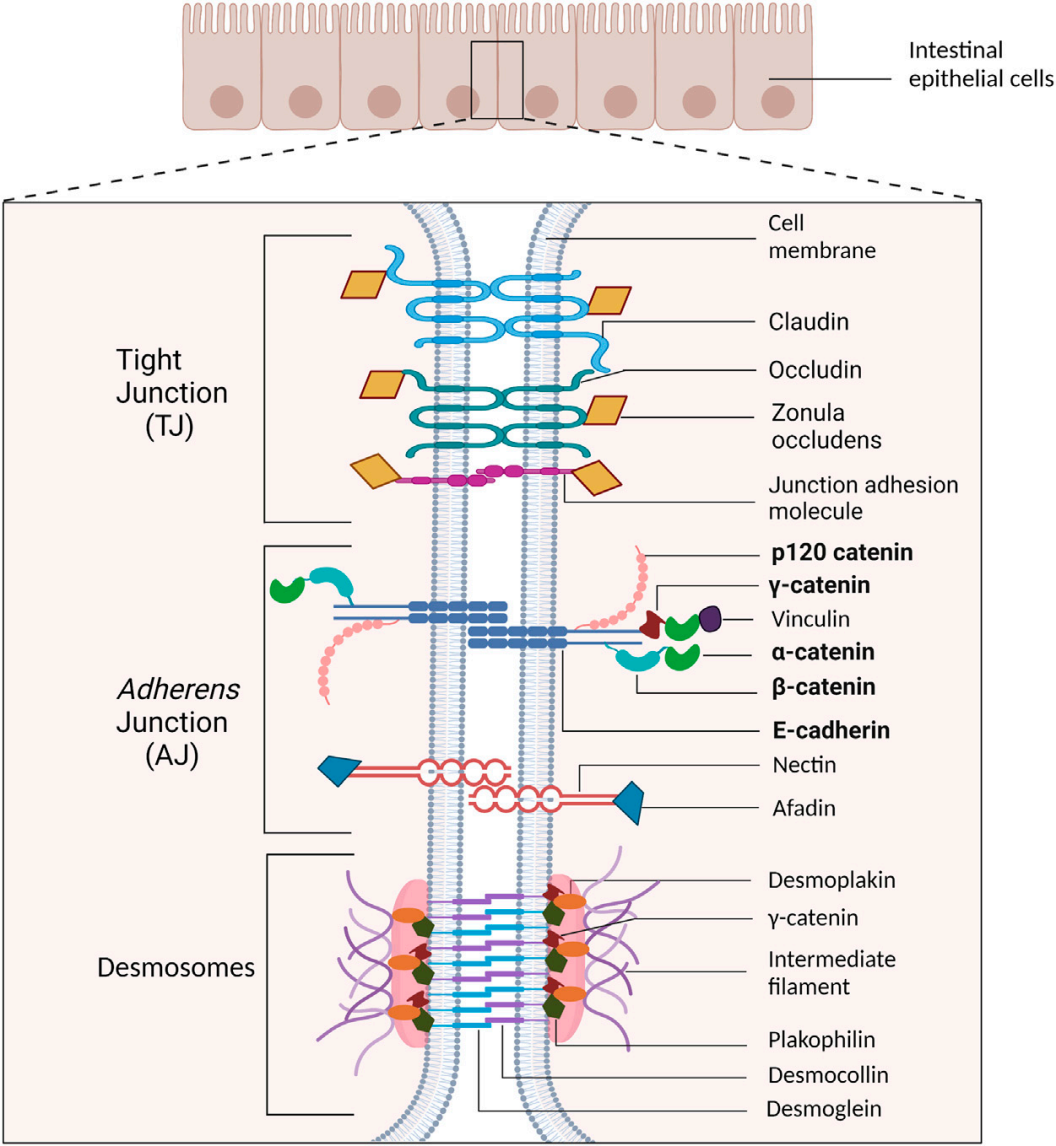
Schematic representation of cell-cell adhesion complexes in intestinal epithelial cells. The tight junction (TJ) is the most apical cell adhesion complex and functions to establish polarity and regulate paracellular permeability. Immediately basal to the TJ is the *adherens* junction (AJ), which plays a key role in mediating cell-cell adhesion. Desmosomal complexes are basal to AJ and are key for strengthening cell-cell adhesion and withstanding mechanical stress. The proteins emphasized in this review are bolded.

Similar to other epithelial cell types, the IEC basolateral membrane consists of an intricate network of multi-protein complexes that regulate paracellular permeability *via* tight junctions (Landy et al., 2016), and cell-cell adhesion *via adherens* junctions (AJs) and desmosomes (Bondow et al., 2012; Mehta et al., 2015; Spindler et al., 2015; Ungewiß et al., 2017). As the most apical cell-cell adhesion complex, tight junctions contribute to maintaining cell polarity. Their primary role, however, is to establish the epithelial cell barrier and regulate the paracellular passage of ions, water, and macromolecules (Hartsock and Nelson, 2008; Ramena and Ramena, 2018). The AJ is located basal to tight junctions and plays a crucial role in mediating cell-cell adhesion (Hartsock and Nelson, 2008; Ramena and Ramena, 2018). Desmosomes, the basal-most junctional complex, provide structural support to the epithelial layer (Ramena and Ramena, 2018), and further strengthens cell–cell adhesion (Raya-Sandino et al., 2021) (Figure 1).

Collectively, cell-cell adhesion complexes regulate paracellular permeability in part by limiting passage of ions and molecules. They also restrict translocation of microbes from the gut lumen into the underlying *lamina propria,* thus serving as a physical barrier that participates in the innate immunity in the intestine (Ali et al., 2020). Indeed, prolonged intestinal barrier disruption leads to increased epithelial permeability and increases the risk of intestinal diseases such as inflammatory bowel disease (IBD) (Lechuga and Ivanov, 2017). Notably, chronic long term intestinal inflammation, concomitant with disruption of cell-cell adhesion, is a major risk factor for the development of colorectal cancer (Rubin et al., 2012; Bujko et al., 2015).

Apart from their roles mediating cell-cell adhesion at the plasma membrane, AJ proteins also have distinct nuclear functions where they participate in cell signaling and transcriptional regulation (van Hengel et al., 1999; Giannini et al., 2000; Kolligs et al., 2000; Cong et al., 2003; Suh and Gumbiner, 2003; Kelly et al., 2004; Reynolds and Roczniak-Ferguson, 2004; Daniel, 2007; Nagel et al., 2010; Valenta et al., 2012; Daugherty et al., 2014; Sun et al., 2014; Su et al., 2015; Aktary et al., 2017; Nagel et al., 2017; Serebryannyy et al., 2017; Zhao et al., 2019). Specifically, nuclear AJ proteins have been shown to interact with transcription factors such as TCF/LEF1 (Maeda et al., 2004; Shang et al., 2017) and Kaiso (Daniel et al., 2002; Jones et al., 2012; Liu et al., 2014) both of which are implicated in regulating canonical Wnt signaling and several human cancers including colorectal cancer (Valenta et al., 2012; Pierre et al., 2019). Interestingly, Kaiso was initially discovered as a binding partner of the catenin p120^ctn^ before it’s role in AJ was fully elucidated (Daniel and Reynolds, 1999; Pierre et al., 2019). In fact, studies to characterize Kaiso as a p120^ctn^ binding partner were pursued because of the first report that β-catenin interacted directly with TCF/LEF1 and localized to the nucleus (Behrens et al., 1996). That seminal publication linking β-catenin and TCF/LEF1 was the first hint that membrane-associated catenin cofactors shuttled in and out of the nucleus to possibly regulate transcription and gene expression. Together the findings of the Birchmeier and Reynolds labs (β-catenin and p120^ctn^ interacting with nuclear transcription factors) laid the foundation for the past two decades of studies seeking to understand how misexpression and/or nucleocytoplasmic localization of AJ proteins contribute to human developmental disorders and cancers.

In this review we discuss cell-cell adhesion complexes in the intestinal epithelial layer with a focus on the nuclear functions of *adherens* junction proteins, their interaction with the transcription factor Kaiso, their impact on cell signaling pathway regulation, and their potential roles in intestinal homeostasis and disease.

### *Adherens* junction proteins in the epithelial cell layer

AJs are comprised of cadherin-catenin complexes that mediate cell-cell adhesion and are anchored to the Actin cytoskeleton (Hartsock and Nelson, 2008). The core component of AJs is the transmembrane protein, epithelial cadherin (E-cadherin) (Hartsock and Nelson, 2008). E-cadherin initiates intercellular contact by forming calcium-dependent homophilic interactions with E-cadherin proteins on adjacent cells (Hartsock and Nelson, 2008; Solanas and Batlle, 2011; Maître and Heisenberg, 2013), and are anchored within the cytoplasm *via* interactions with cofactors called catenins (Yamada et al., 2005; Hartsock and Nelson, 2008). Most of the catenins in the AJ are members of the Armadillo protein family (β-, γ- and p120-catenin) (van Hengel et al., 1999; McCrea and Gottardi, 2016). Armadillo proteins possess several copies of a repeating 42-amino acid motif that facilitates protein-protein interaction and their participation in many essential cellular functions such as nucleocytoplasmic trafficking, transcriptional regulation, and ubiquitination (Tewari et al., 2010).

β-catenin binds the E-cadherin catenin-binding domain *via* its central Armadillo repeat region (Hartsock and Nelson, 2008; Tewari et al., 2010) and facilitates the connection between the AJ complexes and the Actin cytoskeleton through another molecule, α-catenin (Orsulic et al., 1999). Notably, loss of β-catenin in the intestine does not lead to cell-cell adhesion defects (Fevr et al., 2007). Instead, intestines lacking β-catenin display crypt loss and differentiation defects due to the loss of β-catenin’s signaling role as the downstream effector of canonical Wnt signaling (Fevr et al., 2007). This is in contrast to E-cadherin-null intestines that exhibit defects in cell polarity, cell-cell and cell-matrix adhesions, and an increase in paracellular permeability (Schneider et al., 2010), thus further supporting the critical role of E-cadherin in cell-cell adhesion (Tunggal et al., 2005).

Unlike β-catenin that functions to anchor E-cadherin to the Actin cytoskeleton, p120^ctn^ binds E-cadherin at the juxtamembrane domain (Yap et al., 1998; Hartsock and Nelson, 2008; Hu, 2012) and regulates E-cadherin stability and turnover (Ireton et al., 2002; Davis and Reynolds, 2006; Xiao et al., 2007). Similar to E-cadherin-null intestines (Schneider et al., 2010), intestinal-specific p120^ctn^ depletion in mice leads to cell-cell adhesion defects that result in mucosal erosion and terminal intestinal bleeding into the lumen (Smalley-Freed et al., 2010). Moreover, p120^ctn^ ablation in murine intestines reduces E-cadherin and β-catenin levels resulting in an epithelial barrier defect (Smalley-Freed et al., 2010).

Another Armadillo catenin involved in AJ function is γ-catenin. Like β-catenin, γ-catenin also functions to establish a bridge between E-cadherin and α-catenin (Aktary et al., 2017). While not considered an essential component of AJ *per se* (Aktary et al., 2017), Lewis et al. showed that γ-catenin plays a vital role in the stability of these adhesive complexes as epithelial cells lacking both γ-catenin and E-cadherin were unable to form AJs. Reintroduction of E-cadherin only, which facilitated the formation of AJs, was insufficient to stabilize the AJs. However, reintroduction of both γ-catenin and E-cadherin resulted in AJ stabilization and facilitated the organization of desmosomal junctions (Aktary et al., 2017; Lewis et al., 1997). Interestingly γ-catenin is also found in desmosomal junctions where it binds to desmosomal cadherins Desmocollin and Desmoglein. However, γ-catenin’s interaction with α-catenin and the desmosomal cadherins appear to be mutually exclusive as there is partial overlap of the binding sites (Chitaev et al., 1998; Zhurinsky et al., 2000).

Unlike β, γ and p120^ctn^, α-catenin does not have Armadillo repeats (Hulpiau et al., 2013; McCrea and Gottardi, 2016). Instead, α-catenin contains vinculin homology domains and thus does not bind directly to E-cadherin (Herrenknecht et al., 1991; Maiden and Hardin, 2011). Nevertheless, α-catenin functions to connect and anchor the cadherin-catenin complex to the Actin cytoskeleton. α-catenin binds to β-catenin and F-Actin *via* its N- and C-terminus, respectively, thereby stabilizing the AJ and ensuring tissue integrity (Maiden and Hardin, 2011). Loss of α-catenin thus leads to defects in cell adhesion and has been shown to promote cancer development (Benjamin and Nelson, 2008). Collectively, these studies highlight that α-catenin is necessary for the adhesive role of cadherins (Benjamin and Nelson, 2008; Maiden and Hardin, 2011).

Vinculin, another Actin-binding protein found in cell–matrix adhesions and *adherens* junctions, binds to both α- and β-catenin, links cell adhesion complexes to the Actin cytoskeleton and functions in the maintenance of the AJ’s integrity (Peng et al., 2010; Peng et al., 2011). However, Peng et al. (2010) showed that only the binding between β-catenin and Vinculin, and not α-catenin, is crucial for Vinculin’s effect on the organization of AJ. They also showed that Vinculin regulates surface E-cadherin expression through its interaction with β-catenin. While loss of Vinculin did not affect the total levels of E-cadherin, there was reduced E-cadherin expression at the cell membrane which led to decreased cell-cell adhesion. The rescue of cell adhesion in this model by the addition of Vinculin underscores the importance of Vinculin in AJs (Peng et al., 2010).

Nectin-afadin complexes can also be found in AJ where they are known to participate in AJ organization and formation (Takai and Nakanishi, 2003; Takai et al., 2006; Meng and Takeichi, 2009). Afadin is an Actin filament-binding protein that binds to Nectin’s cytoplasmic tail and connects it to the Actin cytoskeleton (Takai et al., 2006; Takai et al., 2008a). Afadin also promotes the association of Nectins with other cell-cell adhesion molecules like E-cadherin *via* its interaction with α-catenin (Tachibana et al., 2000; Takai et al., 2006; Takai et al., 2008a; Takai et al., 2008b). Like E-cadherin, Nectin is a Ca^2+^-independent transmembrane cell-cell adhesion molecule that forms homophilic and heterophilic interactions between neighbouring cells (Takai and Nakanishi, 2003; Takai et al., 2006; Takai et al., 2008a; Okumura et al., 2018). During AJ formation Nectin regulates the rate of assembly of individual AJ components. At the initial stages of cell-cell contact, Nectin from neighbouring cells trans-dimerizes to generate clusters at these contact sites (Takai et al., 2006). These Nectin clusters then recruit E-cadherin, which also trans-dimerizes and results in the formation of E-cadherin clusters. The Nectin- and E-cadherin clusters eventually fuse and develop into mature AJ (Takai et al., 2006; Takai et al., 2008b). The importance of the Nectin-Afadin network in AJ formation was highlighted when Afadin knockout resulted in the loss of organization of cell-cell junctions and failure of cadherin-based AJ formation (Ikeda et al., 1999; Takai et al., 2008b). Importantly, when the homo- and hetero-trans-dimer of Nectin was inhibited, cadherin-based AJ formation did not occur (Takai et al., 2008a).

Once these AJ complexes bind to Actin, Actin binds to α-actinin, which crosslinks the Actin filaments to create a scaffold that stabilizes the attached junctions. α-actinin thus establishes a connection between the cytoskeleton and transmembrane proteins found in the AJ (Otey and Carpen, 2004; Sjöblom et al., 2008).

### Pathologies resulting from faulty cell adhesion in intestinal epithelium

Loss of apical junctional complexes perturbs cell-cell adhesion and compromises intestinal barrier integrity. This was first demonstrated by Hermiston and Gordon using their chimeric transgenic mice that expressed dominant negative N-cadherin (NCADΔ) (Hermiston and Gordon, 1995). N-cadherin is a Ca^2+^-dependent adhesion molecule that functions like E-cadherin to mediate cell–cell adhesion in AJ (Van Patten et al., 2010). NCADΔ mice exhibit reduced endogenous E-cadherin, a defective intestinal epithelial barrier and increased infiltration of bacteria (Hermiston and Gordon, 1995). Similarly, intestinal-specific E-cadherin knockout (*VilCre:Cdh1* ^*loxP/loxP*^) causes perinatal death that is likely due to an impaired epithelial barrier. Indeed, by embryonic day 18.5, *VilCre:Cdh1* ^*loxP/loxP*^ mice already exhibit gaps in the intestinal barrier and deterioration of the epithelium (Bondow et al., 2012). Consistent with these findings, inducible E-cadherin inactivation in adult mice also caused disintegration of the intestinal epithelium (Schneider et al., 2010).

It is well established that IBD patients exhibit enhanced intestinal permeability (Hollander, 1999; Secondulfo et al., 2001; Vivinus-Nébot et al., 2014) which is attributed in part to defective intercellular adhesion (Lechuga and Ivanov, 2017). Indeed, inflamed intestinal tissues in both humans and rodent models of IBD exhibit misexpression of several adhesion-associated proteins, a finding supported by genome-wide association studies of both Crohn’s disease (CD) and ulcerative colitis (UC), reviewed in (Mehta et al., 2015). Interestingly, in inflamed intestinal mucosa of both active CD and UC, a phenomenon known as cadherin switching, where the expression of E-cadherin decreases and the expression of P-cadherin increases, is observed (Sanders et al., 2000; Naydenov et al., 2022). While E-cadherin and P-cadherin are both classical cadherins and expressed in epithelial tissues, E-cadherin is the most highly expressed in healthy epithelial tissues in the gastrointestinal tract (Sanders et al., 2000), while P-cadherin is poorly expressed (Naydenov et al., 2022). Moreover, E-cadherin and P-cadherin have different adhesive properties and participate in different signaling activities, and thus the functional relevance of this switching in the inflamed intestinal mucosa remains to be determined (Naydenov et al., 2022). Regardless, the *CDH1* and *CDH3* loci, which encode E-cadherin and P-cadherin respectively, are among the various susceptibility loci implicated in IBD (Sanders et al., 2000; Barrett et al., 2009; Mehta et al., 2015).

Given p120^ctn^’s role in stabilizing E-cadherin at the AJ, it is not surprising that loss of p120^ctn^ has also been associated with intestinal inflammation (Perez-Moreno et al., 2006; Perez-Moreno et al., 2008; Smalley-Freed et al., 2010). Intestinal-specific loss of p120^ctn^ resulted in a concomitant reduction in E-cadherin and impaired cell-cell adhesion (Smalley-Freed et al., 2010). Moreover, p120^ctn^ knockout mice also exhibited histological features of IBD, including crypt hyperplasia, cell-cell adhesion defects, mucosal thickening and increased neutrophil infiltration (Smalley-Freed et al., 2010). Similar findings were also observed in tamoxifen-induced p120^ctn^ mosaic knockout mice, which developed intestinal adenomas as they aged (Smalley-Freed et al., 2011).

Interestingly, colon cancer usually presents with altered gene expression of many cell-cell adhesion proteins, which perturbs cell-cell adhesion and intestinal permeability, and enhances the invasive ability of colon cancer (Tsanou et al., 2008; Bujko et al., 2015). Indeed, reduced localization of β-catenin at the AJ, concomitant with its translocation to the nucleus activates transcription of tumour-promoting genes such as *cyclin D1*, *matrilysin*, and *c-myc* (Wong and Pignatelli, 2002). Collectively, these findings highlight the importance of functional apical junctional complexes in maintaining the normal physiology and homeostasis of the mammalian intestine and how their malfunction contributes to disease.

The fundamental role that membrane-associated cadherin and catenin proteins play in establishing and maintaining intestinal tissue integrity is well documented (Benjamin and Nelson, 2008; Schneider et al., 2010; Smalley-Freed et al., 2010; Schneider and Kolligs, 2015; Aktary et al., 2017). However, their nuclear roles and impacts on transcriptional regulation and epithelial barrier disruption are still being elucidated and are an emerging research area. Below, we summarize findings on the AJ proteins with characterized nuclear functions and their implications for intestinal diseases.

### Nuclear roles of *adherens* junction proteins

Recently, many published studies have highlighted a nuclear role for several AJ proteins, namely E-cadherin (Su et al., 2015; Zhao et al., 2019), β-catenin (Cong et al., 2003; Suh and Gumbiner, 2003; Valenta et al., 2012), γ-catenin (Kolligs et al., 2000; Nagel et al., 2010; Aktary et al., 2017; Nagel et al., 2017), α-catenin (Giannini et al., 2000; Daugherty et al., 2014; Sun et al., 2014; Serebryannyy et al., 2017), and p120^ctn^ (van Hengel et al., 1999; Kelly et al., 2004; Reynolds and Roczniak-Ferguson, 2004; Daniel, 2007). These nuclear functions are linked to transcriptional regulation that ultimately contributes to intestinal diseases such as IBD and colon cancer (van Hengel et al., 1999; Giannini et al., 2000; Kolligs et al., 2000; Cong et al., 2003; Suh and Gumbiner, 2003; Kelly et al., 2004; Reynolds and Roczniak-Ferguson, 2004; Daniel, 2007; Nagel et al., 2010; Valenta et al., 2012; Daugherty et al., 2014; Sun et al., 2014; Su et al., 2015; Aktary et al., 2017; Nagel et al., 2017; Serebryannyy et al., 2017; Zhao et al., 2019). While many studies have reported that the nuclear functions of AJ proteins are distinct from their roles at the plasma membrane, nuclear localization of AJ proteins often coincides with a concomitant loss of expression at the epithelial cell membrane (Hao et al., 2001; El-Bahrawy et al., 2002; Daniel, 2007; Su et al., 2015). This suggests that nuclear AJ proteins may be a marker of intestinal disease.

### Nuclear E-cadherin modifies canonical Wnt signaling

In addition to catenin cofactors, the transmembrane protein E-cadherin interacts with several additional proteins of varying functions—these include GTPase regulators, kinases, phosphatases, and Actin dynamics regulators to name a few (Guo et al., 2014; Van Itallie et al., 2014). While some of these E-cadherin binding partners are localized at cell-cell junctions (e.g., Tropomodulin-3) some were found to localize in the cytoplasm (e.g., Filamin-A), within or around the nucleus (e.g., Ran GTPase-activating protein 1) and in vesicles (e.g., Retinoic acid-induced protein 3 (reviewed in Guo et al., 2014). Interestingly, most of the proteins involved in regulating Actin dynamics or function as adaptors localize at cell junctions, in contrast to those involved in transcription, translation and metabolism (Guo et al., 2014). These findings suggest that E-cadherin itself localizes in multiple subcellular compartments (cell membrane, cytoplasm, nucleus), to facilitate its binding to diverse proteins located in distinct subcellular compartments.

Indeed, nuclear localization of E-cadherin has been reported in several cell types, including lung and colon cancer cells (Su et al., 2015; Zhao et al., 2019). Zhao et al. (2019) found an enrichment of E-cadherin in the nuclei of HCT116 and SW480 colon cancer cells and in human colon cancer tissues concomitant with a loss of membrane localization. They also showed that E-cadherin nuclear translocation occurs in a p120^ctn^-dependent manner as E-cadherin does not possess a nuclear localization signal (NLS) (Zhao et al., 2019). They also showed that p120^ctn^ overexpression enhanced E-cadherin’s nuclear localization in HCT116 colon cancer cells (Zhao et al., 2019). Interestingly, Ferber *et al.* also found that p120^ctn^ enhanced the nuclear translocation of E-cadherin’s cleaved cytoplasmic domain (Ferber et al., 2008). E-cadherin’s intracellular domain is cleaved by γ-secretase, releasing the E-cadherin C-terminal fragment 2 (E-cad/CTF2) that then translocates into the nucleus where it regulates transcription. While E-cad/CTF2 can bind DNA, this binding is conditional upon it first binding to p120^ctn^. When bound to p120^ctn^, E-cad/CTF2 enhances p120^ctn^'s inhibitory transcriptional effects. E-cad/CTF2 is also implicated in apoptosis, however the exact mechanism has yet to be determined (Ferber et al., 2008).

**FIGURE 2:**
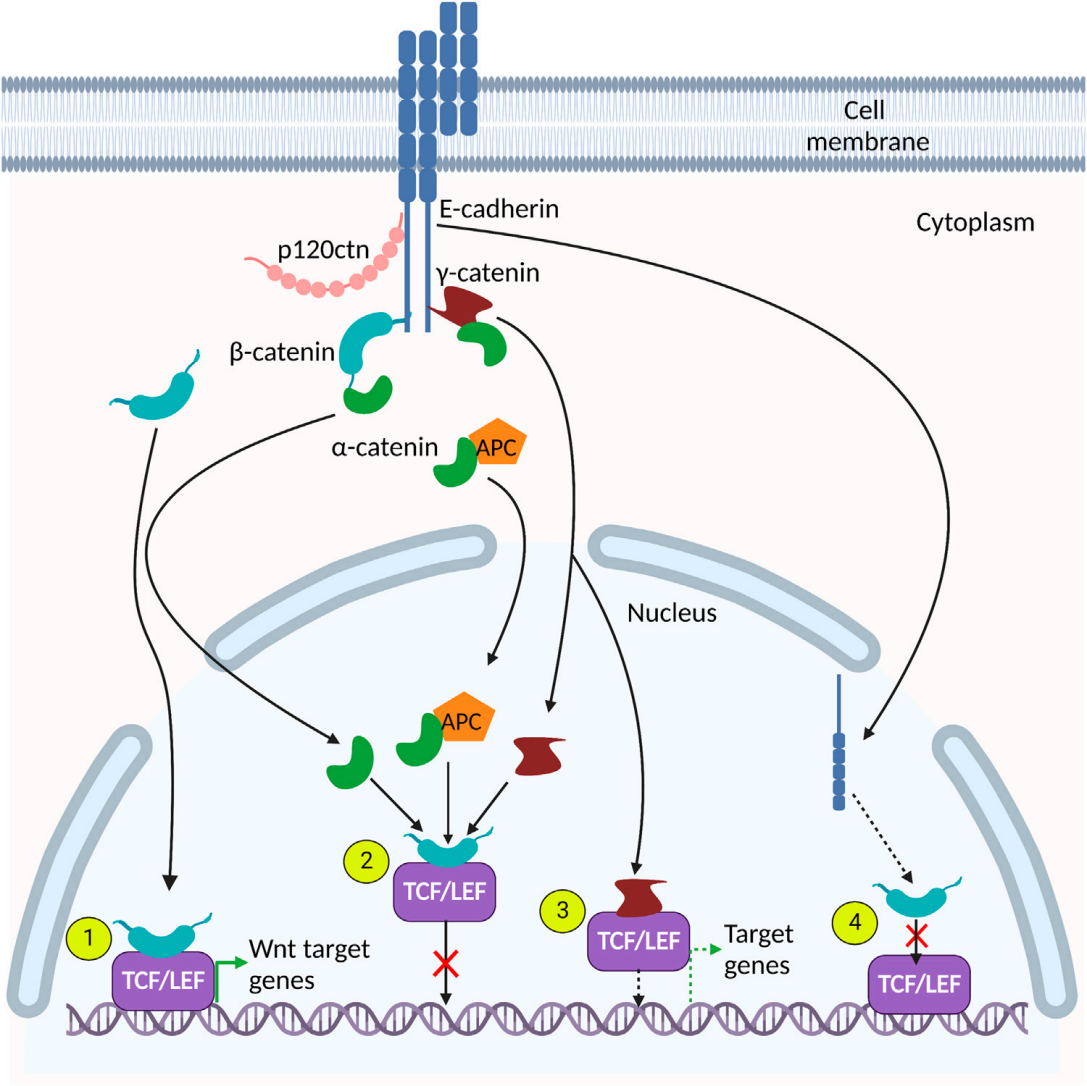
*Adherens* junction (AJ) protein interactions in the nucleus. Many AJ proteins interact with components of the canonical Wnt signaling pathway. (1). β-catenin interacts directly with TCF/LEF to activate Wnt target genes. (2). α-catenin, γ-catenin and α-catenin/APC complexes interact with the β-catenin-TCF/LEF heterodimer and prevents its association with DNA, thereby inhibiting expression of Wnt target genes. (3). γ-catenin binds directly to TCF/LEF and activates TCF/LEF-dependent gene expression. (4). E-cadherin interacts with β-catenin and disrupts its interaction with TCF/LEF cofactors.

E-cadherin nuclear localization and function can also be regulated by post-translational modifications (Su et al., 2015; Zhao et al., 2019). For example, Su et al. (2015) showed that Src kinase-mediated tyrosine phosphorylation of E-cadherin in cancer stem cells resulted in E-cadherin endocytosis and nuclear translocation. In the nucleus, E-cadherin competed with TCF4 for binding to β-catenin’s Armadillo repeat domain, thus inhibiting β-catenin/TCF4 heterodimerization (Su et al., 2015) (Figure 2 and Table 1 described in more detail in the following section). Subsequently, there was a decrease in canonical Wnt-mediated signaling and metastasis by cancer stem cells (Su et al., 2015). In addition to tyrosine phosphorylation, nuclear E-cadherin can also be acetylated on lysine residues *via* the acetyltransferase CREB-binding protein (CBP) (Zhao et al., 2019). This prevents protein interactions between E-cadherin and β-catenin, therefore potentiating β-catenin’s transcriptional and oncogenic activities in HCT116 cells. Importantly, increased acetylated E-cadherin correlated with poorer clinical outcomes in colorectal cancers (Zhao et al., 2019). Collectively, these findings highlight a role for nuclear E-cadherin in colorectal tumorigenesis and metastasis (Zhao et al., 2019).

**TABLE 1:** Summary of the interactions of nuclear *adherens* junction proteins.

AJ protein	Nuclear interactor(s)	Consequences of AJ protein nuclear localization	Reference(s)
E-cadherin	Nuclear β-catenin	Reduces transcriptional activity of β-catenin/TCF4 complexes by binding to β-catenin, preventing it from binding to TCF4	(Su et al., 2015; Zhao et al., 2019)
β-catenin	TCF/LEF complex	Initiates expression of Wnt target genes (e.g., *c-myc* and *cyclin D1*) by providing a transactivation domain to TCF/LEF.	(Valenta et al., 2012; Shang et al., 2017; Daniels and Weis, 2005; Kolligs et al., 1999; Tetsu and McCormick, 1999; Behrens et al., 1996)
γ -catenin	β-catenin-TCF/LEF complex	Prevents β-catenin-TCF/LEF DNA-binding and attenuates Wnt signaling	(Miravet et al., 2002; Aktary et al., 2017)
	TCF/LEF complex	Activates TCF/LEF-dependent transcription	(Maeda et al., 2004; Kolligs et al., 2000)
α-catenin	β-catenin-TCF/LEF complex	Prevents β-catenin-TCF/LEF DNA-binding and attenuates Wnt signaling	(Giannini et al., 2000; Daugherty et al., 2014; Sun et al., 2014)
p120 catenin	Kaiso	Prevents Kaiso DNA-binding and inhibits Kaiso’s transcriptional activities	(Daniel and Reynolds, 1999; Daniel et al., 2002; Kelly et al., 2004; Daniel, 2007; del Valle-Pérez et al., 2016; McCrea and Gottardi, 2016)

The studies above highlight the versatility of E-cadherin and the possibility for both membrane-bound E-cadherin and E-cad/CTF2 to contribute to intestinal diseases and cancer. Additional studies are needed to determine the mechanism by which E-cadherin and E-cad/CTF2 translocate into the nucleus and to ascertain whether the p120^ctn^ NLS plays a role in this process. Moreover, given that nuclear E-cadherin can modulate β-catenin’s transcriptional and oncogenic activities, studies to further understand this interaction and its implications for intestinal diseases are needed.

### Nuclear β-catenin is a key downstream effector of the canonical Wnt signaling pathway

Of all the catenins involved in the AJ, β-catenin is the most studied and characterized with respect to its nuclear roles. Nuclear accumulation of β-catenin is a key event in canonical Wnt signaling (Shang et al., 2017; Tanaka et al., 2019; Cheng et al., 2019), which is fundamental to maintain the intestinal stem cell niche and integral to IEC differentiation. Importantly, ∼ 90% of colorectal cancers exhibit dysregulated Wnt signaling (Fevr et al., 2007).

β-catenin can be found in four distinct subcellular locations––at the plasma membrane in cell-cell contacts, in the cytoplasm, in the nucleus and at centrosomes (Mbom et al., 2013; Kam and Quaranta, 2009; Bienz, 2004). The membrane-bound pool is stabilized by binding to E-cadherin where it functions to anchor E-cadherin to the Actin cytoskeleton (Bienz, 2004). However, newly synthesized β-catenin and β-catenin released from the AJ—which makes up the cytoplasmic pool—are normally unstable and short-lived (Valenta et al., 2012). In the absence of Wnt signaling, the free cytoplasmic pool of β-catenin is phosphorylated by a destruction complex, comprised of Adenomatous polyposis coli (APC), Axin, Casein kinase 1 (CK1) and Glycogen synthase kinase 3 (GSK3), that targets cytoplasmic β-catenin for proteasomal degradation (Cong et al., 2003; Valenta et al., 2012; Shang et al., 2017). However, upon activation of the canonical Wnt pathway, β-catenin escapes degradation, accumulates in the cytoplasm and then translocates into the nucleus (Cong et al., 2003; Suh and Gumbiner, 2003; Valenta et al., 2012; Arnold et al., 2020). Nuclear translocation of β-catenin occurs independently of importin-β and Ran GTPase, proteins essential for nuclear transport. While β-catenin does not possess an NLS, it was shown to interact directly with nuclear pore proteins *via* its Armadillo domain and it was postulated that this interaction facilitates its nuclear translocation (Fagotto et al., 1998; Yokoya et al., 1999; Morgan et al., 2014; Anthony et al., 2020). Sharma et al., (2016) also highlighted a role for the hydrophobic amino acids in the N- and C- terminal regions of β-catenin in mediating its nuclear translocation. However, β-catenin was also suggested to utilize NLS-containing chaperone proteins such as BCL9, APC and LEF to facilitate its nuclear translocation (Morgan et al., 2014; Anthony et al., 2020). Recently however, in colon cancers carrying mutations in *APC*, a member of the importin-β family, IPO11, was found to interact with β-catenin in a Ran-dependent manner, mediating its import into the nucleus (Mis et al., 2020). Interestingly, IPO11-dependent nuclear translocation of β-catenin was not observed in colon cancer cells with wildtype *APC* (Mis et al., 2020). These findings suggest that the mechanisms whereby β-catenin translocates to the nucleus is complex and context-dependent (Mis et al., 2020).

The tumor suppressor APC interacts with β-catenin in two distinct sub-cellular locations—in the cytoplasm and the nucleus (Hankey et al., 2018). In the cytoplasm APC is important for the degradation of β-catenin (Munemitsu et al., 1995; Yang et al., 2006) while in the nucleus, it negatively regulates the transcriptional activity of β-catenin (Rosin-Arbesfeld et al., 2003; Xiong and Kotake, 2006), and facilitates its nuclear export and subsequent cytoplasmic degradation (Henderson, 2000; Xiong and Kotake, 2006; Hankey et al., 2018). When APC is mutated, it is still capable of binding to β-catenin (Henderson, 2000), and Yang et al. (2006) showed that β-catenin phosphorylation also still occurs. However, Ikeda et al. (1998) showed that phosphorylation of β-catenin also occurs in APC-deficient cells, suggesting that APC does not regulate β-catenin phosphorylation. Studies aimed at further elucidating APC’s function show that APC is necessary for the ubiquitination of β-catenin (Yang et al., 2006; Su et al., 2008) since mutation of APC prevented β-catenin ubiquitination and its subsequent degradation, and led to its nuclear accumulation (Yang et al., 2006).

Once in the nucleus, β-catenin binds TCF/LEF1 transcription factors, which on their own, lack transcriptional activity (Shang et al., 2017). However, the β-catenin/TCF/LEF1 complex forms a functional heterodimer that modulates transcription and upregulates the expression of Wnt target genes (Cong et al., 2003; Suh and Gumbiner, 2003; Valenta et al., 2012) (Figure 2 and Table 1). Interestingly, mutated APC can still enter the nucleus where it binds to and exports β-catenin from the nucleus, thus maintaining the nuclear export function of APC (Henderson, 2000). Collectively, these studies suggest that mutated APC can affect the transcriptional activity of β-catenin but not its expression levels (Henderson, 2000). In this review, we focus on the nuclear roles of β-catenin in mammalian intestines. For additional details on β-catenin nuclear interactions see reviews (Anthony et al., 2020; McCrea and Gottardi, 2016) and (Valenta et al., 2012).

In the intestine, canonical Wnt signaling plays a critical role in the maintenance and renewal of the intestinal epithelium by regulating stem cell proliferation and differentiation (Mah et al., 2016; Perochon et al., 2018). Since nuclear β-catenin is the key effector of canonical Wnt signaling, it plays a key role in intestinal homeostasis. Wnt/β-catenin-mediated transcription maintains many aspects of tissue homeostasis, including cell renewal (maintenance of stemness and the undifferentiated state of intestinal stem cells), and intestinal regeneration after injury (Fevr et al., 2007; Shang et al., 2017), both of which are characterized by increased proliferation within the crypt compartment (Perochon et al., 2018). This is accomplished specifically *via* the activation of Wnt target genes involved in cell proliferation (e.g., *c-myc*, *cyclin D1*). Wnt/β-catenin signaling also contributes to tissue homeostasis *via* regulation of the intestinal lineage differentiation of transit amplifying cells. (Fevr et al., 2007; Shang et al., 2017).

Elevated canonical Wnt signaling, and therefore increased nuclear β-catenin, perturbs intestinal homeostasis and increases the risk of colon cancer (Shang et al., 2017). *In vitro* and *in vivo* models of colorectal neoplasia displayed nuclear localization of β-catenin (Sheng et al., 1998). Nuclear β-catenin was also observed in aberrant crypt foci, which are early neoplastic lesions that precede malignant transformation in the colon (Hao et al., 2001). This finding suggests that aberrant localization of β-catenin is an early event in colorectal tumorigenesis (Sheng et al., 1998; Hao et al., 2001), a concept further strengthened by Sheng et al. (1998) who noted that nuclear β-catenin is a common occurrence in intestinal adenomas. Studies have also found that β-catenin localizes primarily to the plasma membrane in normal colon tissues, but exhibits decreased membrane association and enhanced nuclear localization in intestinal adenomas and carcinomas (Hao et al., 2001; Chen et al., 2008; Stanczak et al., 2011). Indeed, elevated levels of nuclear β-catenin has been observed in ∼ 80% of colorectal cancers (White et al., 2012; Shang et al., 2017), and this high expression of nuclear β-catenin correlated with poor overall survival (Mårtensson et al., 2007; Chen et al., 2008; Chen et al., 2013; Kim et al., 2019) and poor prognosis (Mårtensson et al., 2007; Chen et al., 2013).

While increased nuclear β-catenin localization due to aberrant canonical Wnt signaling is an early and important alteration in CRC progression (Zhang et al., 2016), genomic mutations in β-catenin also occur, albeit more rarely (Kitaeva et al., 1997; Kim et al., 2019; Arnold et al., 2020). Most mutations occur in exon 3 (Kitaeva et al., 1997; Sparks et al., 1998; Samowitz et al., 1999; Johnson et al., 2005; Kim et al., 2019; Arnold et al., 2020) and involve deletions, transitions or transversions, causing a loss of the serine/threonine phosphorylation sites (Kitaeva et al., 1997; Sparks et al., 1998; Samowitz et al., 1999; Arnold et al., 2020). These mutations allow β-catenin to escape proteasomal degradation (Sparks et al., 1998; Kim et al., 2019), leading to increased cytoplasmic accumulation, subsequent nuclear translocation, and ultimately increased Wnt signaling activity (Kim et al., 2019). β-catenin mutations have also been observed in adenomas suggesting that genomic mutations in β-catenin may also be an early occurrence in colorectal tumorigenesis (Sparks et al., 1998; Samowitz et al., 1999).

### Nuclear γ-catenin modulates CRC tumorigenesis

γ-catenin, also known as Plakoglobin, is another member of the catenin protein family with known nuclear localization and functions (Lifschitz-Mercer et al., 2001; Nagel et al., 2010; Lombardi et al., 2011; Aktary et al., 2013). Nuclear γ-catenin plays a role in biological processes such as adipogenesis and fibrogenesis (Garcia-Gras et al., 2006; Lombardi et al., 2011). Nuclear γ-catenin also interacts with and enhances the transcriptional activity of both the wild type and mutant form of p53, highlighting a role in regulating gene expression (Aktary et al., 2013). Protein interactions between γ-catenin and p53 have been observed in both the cytoplasm and nucleus. In the nucleus, γ-catenin/p53 complexes induced expression of 14-3-3σ, which negatively regulates cell cycle and positively regulates the transcriptional activity of p53 (Aktary et al., 2013).

Whether nuclear γ-catenin promotes or prevents CRC progression remains unresolved. For example, Lifschitz-Mercer et al. (2001) found more nuclear γ-catenin in normal compared to neoplastic human intestinal tissue samples. However, in a study by Nagel et al. (2010) strong nuclear γ-catenin localization was observed in invasive CRC samples relative to normal colon tissues where no nuclear localization was observed. These conflicting findings warrant further characterization of the role that nuclear γ-catenin plays in the malignant transformation and progression of CRC.

The Armadillo domains of γ- and β-catenin share ∼80% amino acid sequence identity and these catenins are sometimes deemed to be paralogs since they are reported to interact with many of the same proteins (Kolligs et al., 2000; Aktary et al., 2017). Indeed, immunoprecipitation of the cytoplasmic and nuclear fractions of γ-catenin-overexpressing HCT116 colorectal carcinoma cells revealed enriched γ-catenin/TCF4 interactions in the nuclear fraction (Pan et al., 2007). Maeda et al. (2004) also detected binding of γ-catenin to both TCF and LEF in immunoprecipitation assays and demonstrated that γ-catenin can alter TCF/LEF-mediated transcriptional regulation (Figure 2 and Table 1). Furthermore, γ-catenin disrupts β-catenin-TCF/LEF complexes, preventing their interaction with DNA and thus attenuating canonical Wnt signaling (Miravet et al., 2002; Aktary et al., 2017) (Figure 2 and Table 1). In agreement with this finding, γ-catenin was found to activate Wnt signaling in DLD-1 cells, and γ-catenin depletion led to decreased tumor cell invasion and increased apoptosis in anchorage-dependent cells (Nagel et al., 2017). Moreover, re-expression of γ-catenin in β-catenin-deficient RKO colon cancer cells resulted in activation of Wnt signaling, but decreased cell proliferation *via* modulation of the cell cycle (Nagel et al., 2017).

Collectively, these findings suggest that γ-catenin’s role in colon tumorigenesis is context-dependent, and that γ-catenin can influence CRC tumorigenesis *via* TCF/LEF-dependent and -independent mechanisms (Aktary and Pasdar, 2012; Aktary et al., 2017). Currently, much of what is known about nuclear γ-catenin’s role in CRC was determined in studies using cultured cell lines. Therefore, additional research using complementary approaches such as intestinal organoids and murine models of colorectal cancer and IBD are needed to fully elucidate the role of nuclear γ-catenin in intestinal disease. Moreover, given that p53 mutations are common in colon cancer (Kameyama et al., 2018) and γ-catenin interacts directly with both wild type and mutant p53, a thorough investigation into the role of these interactions in the context of intestinal homeostasis and disease is warranted. Additional research is also needed to elucidate the mechanism of nuclear translocation of γ-catenin.

### Nuclear α-catenin inhibits tumorigenesis

Numerous studies have reported that α-catenin is capable of trafficking between the cytoplasm and the nucleus (Giannini et al., 2000; El-Bahrawy et al., 2002; Daugherty et al., 2014; Serebryannyy et al., 2017). α-catenin exhibits strong nuclear localization in colon cancer cell lines (Giannini et al., 2000; El-Bahrawy et al., 2002), which was more evident in dispersed rather than confluent cells (El-Bahrawy et al., 2002). This finding suggests that α-catenin nuclear localization is concomitant with a loss of cell-cell adhesive properties. Interestingly, α-catenin showed near mutually exclusive localization between the nucleus and plasma membrane — colorectal adenocarcinoma tissues with significant plasma membrane localization of α-catenin showed minimal nuclear localization and vice versa (El-Bahrawy et al., 2002).

Functionally, loss of α-catenin is implicated in colorectal cancer and cellular dysfunction (Raftopoulos et al., 1998; Cheng et al., 2016). Reduced expression of α-catenin led to increased cell proliferation, possibly due to its indirect interaction with pathways that modulate cell proliferation such as the Wnt signaling pathway (Benjamin and Nelson, 2008; Sun et al., 2014). Notably, α-catenin has been implicated in regulating the canonical Wnt pathway by binding to the β-catenin-TCF complex, disrupting its interaction with DNA (Giannini et al., 2000; Daugherty et al., 2014; Sun et al., 2014; McCrea and Gottardi, 2016; Serebryannyy et al., 2017), and inhibiting transcription of Wnt/β-catenin target genes such as *c-myc* (Daugherty et al., 2014) (Figure 2 and Table 1). α-catenin’s inhibition of Wnt/β-catenin signaling was also demonstrated by Giannini et al. (2000) who reported that α-catenin depletion increased β-catenin-TCF-dependent transcription. Thus, α-catenin may act to fine-tune the canonical Wnt pathway, since its nuclear localization is dependent on nuclear β-catenin, which itself depends on active Wnt signaling (Daugherty et al., 2014). Additionally, studies show that α-catenin also interacts with cytoplasmic APC protein, recruiting it to the β-catenin-TCF complex in the nucleus and suppressing the expression of Wnt target genes (Choi et al., 2013; Sun et al., 2014) (Figure 2).

Interestingly in colon cancer cells, nuclear α-catenin also interacts with nuclear F-Actin (Daugherty et al., 2014; Serebryannyy et al., 2017). Serebryannyy et al. (2017) showed that upon binding to nuclear F-Actin, α-catenin recruitment to sites of DNA damage was enhanced. This interaction between nuclear α-catenin and F-Actin also highlights a possible role for α-catenin in nuclear Actin dynamics. This possibility is further supported by the finding that nuclear α-catenin induces nuclear F-Actin formation which is associated with changes in chromatin organization (Daugherty et al., 2014). This implicates α-catenin as a regulator of gene expression *via* its effects on nuclear Actin organization. Indeed α-catenin’s inhibitory effect on the transcription of Wnt target genes also requires its Actin-binding domains (Daugherty et al., 2014).

Collectively, these findings hint that nuclear α-catenin inhibits tumorigenesis, although the molecular mechanisms involved in the nucleo-cytoplasmic shuttling of α-catenin needs to be further explored. Moreover, additional studies are warranted to fully elucidate whether nuclear α-catenin plays a functional role in CRC progression and whether nuclear translocation of α-catenin is a cause or consequence of intestinal dysfunction.

### Nuclear p120^ctn^ interacts with transcriptional regulators

Aside from its role in providing stability to E-cadherin at the plasma membrane, the Src substrate p120^ctn^ also shuttles in and out of the nucleus (Roczniak-Ferguson and Reynolds, 2003), hinting a nuclear role. This catenin has two classical NLSs—one localized in the phosphorylation domain and the other in Armadillo (Arm) repeat 6 (Roczniak-Ferguson and Reynolds, 2003). However, studies have shown that p120^ctn^ nuclear translocation is mediated *via* alternate sequences in its Arm domain rather than *via* its conventional NLSs (Roczniak-Ferguson and Reynolds, 2003; Daniel, 2007; van Hengel and van Roy, 2007). Roczniak-Ferguson and Reynolds found that p120^ctn^ Arm repeats 3 and 5 are essential for its entry into the nucleus (Roczniak-Ferguson and Reynolds, 2003). However, Kelly et al. (2004) showed that nuclear localization of p120^ctn^ is mediated *via* another highly basic NLS located between Arm repeats 6 and 7. These findings suggest that p120^ctn^ utilizes different mechanisms to shuttle into the nucleus, possibly due to it having many functional isoforms (Kelly et al., 2004). While the functional significance of the p120^ctn^ isoforms generated as a result of alternative splicing are yet to be fully elucidated (Roczniak-Ferguson and Reynolds, 2003), it has been postulated that each p120^ctn^ isoform participates in distinct cellular processes and utilizes different mechanisms to shuttle into the nucleus (Kelly et al., 2004). Intriguingly, using a DNA-cellulose binding assay, Ferber *et al.* showed that nuclear p120^ctn^ can bind directly to DNA (Ferber et al., 2008). If it is experimentally validated that endogenous p120^ctn^ binds directly to DNA, this will add another level of complexity to the role of AJ proteins in regulating transcription, gene expression and tumorigenesis.

Although p120^ctn^ can localize to the nucleus, its nuclear function in intestinal diseases is not as well characterized as its membrane function, or the nuclear functions of β-catenin. However, a few studies have attempted to elucidate the nuclear functions of p120^ctn^ in other pathologies. For example, p120^ctn^ has been implicated in pancreatic cancer where its nuclear localization is associated with tumour malignancy by promoting cancer cell proliferation (Mayerle et al., 2003). Nuclear p120^ctn^ was also found to bind to and relieve repression of the transcriptional regulators RE1-silencing transcription factor (REST) and CoREST, thus activating their target genes (Lee et al., 2014; McCrea and Gottardi, 2016). As REST and CoREST regulate the expression of genes involved in neuronal differentiation, nuclear p120^ctn^ is implicated in neuronal differentiation (Lee et al., 2014; McCrea and Gottardi, 2016). Additionally, p120^ctn^ nuclear localization during junction disintegration in endothelial cells (Beckers et al., 2008) suggests that its nuclear localization may occur as a result of loss of AJ integrity. Thus, the relationship between nuclear p120^ctn^ and intestinal diseases needs further investigation to gain a better understanding of how p120^ctn^'s nuclear roles affect intestinal function and homeostasis, and to discern cause verses consequence.

**FIGURE 3:**
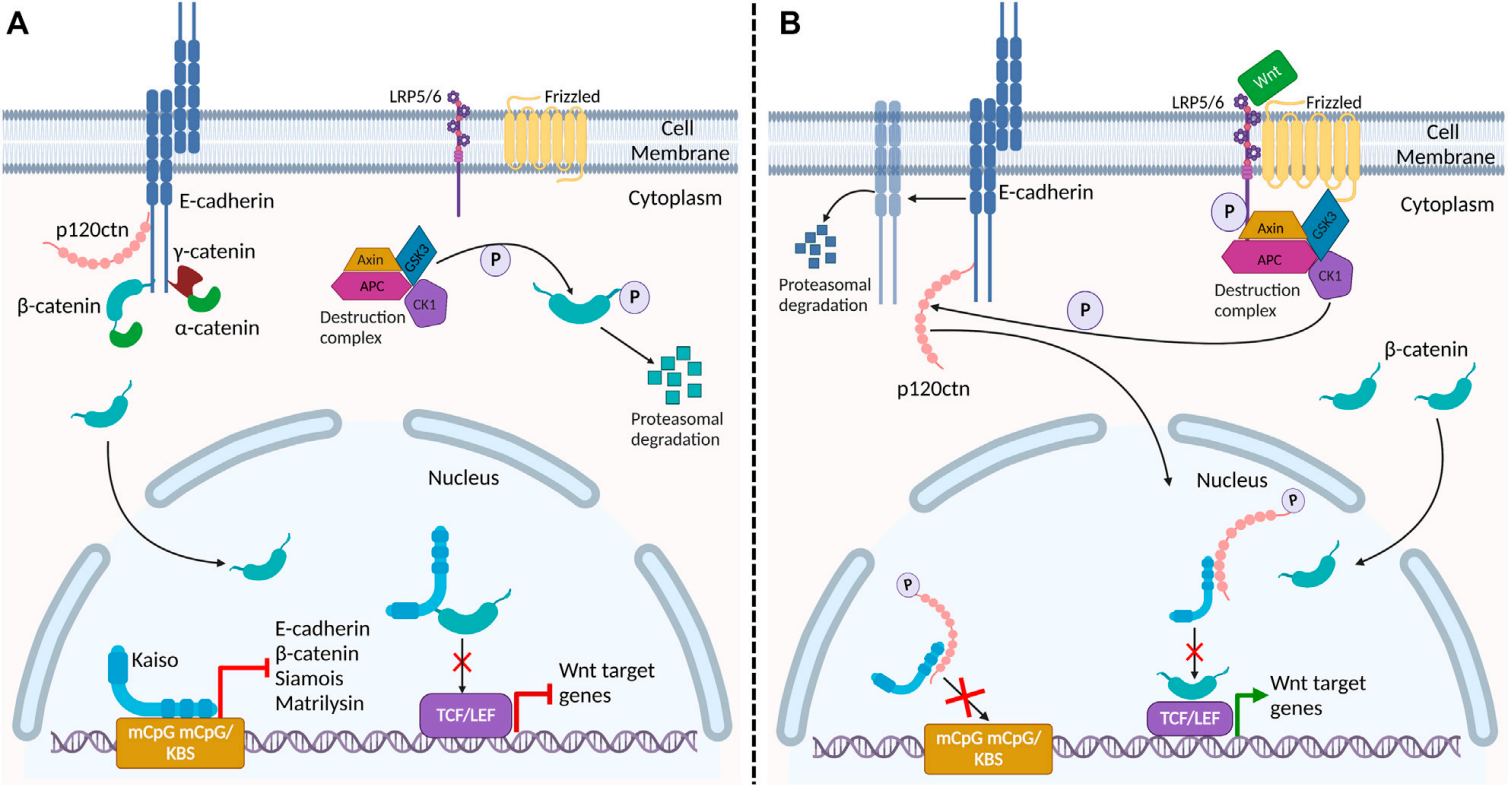
Proposed interaction of *adherens* junction proteins and Kaiso in the Wnt signaling pathway and epithelial barrier stability. **(A)** In the absence of Wnt signaling, β-catenin and p120^ctn^ bind E-cadherin at the membrane, providing stability to E-cadherin at the plasma membrane. Cytosolic β-catenin is phosphorylated and targeted for degradation by the destruction complex. In the nucleus, Kaiso binds to the promoter region of target genes β-catenin (*CTNNB1*), E-cadherin (*CDH1*)*, Siamois* and *matrilysin (mmp7)* repressing their transcription. Kaiso also binds to β-catenin, preventing its interaction with TCF/LEF. **(B)** Activation of the canonical Wnt signaling pathway inactivates the destruction complex, resulting in cytosolic accumulation and nuclear translocation of β-catenin. Wnt signaling also triggers CK1, a component of the destruction complex, to phosphorylate p120^ctn^. Phosphorylated p120^ctn^ then dissociates from E-cadherin and translocates to the nucleus where it relieves Kaiso-mediated regulation of genes. Reduced E-cadherin-associated p120^ctn^ at the plasma membrane results in increased E-cadherin degradation, leading to defective cell adhesion and increased permeability in the epithelial barrier. Given that nuclear p120^ctn^ blocks Kaiso’s DNA binding domain, we propose that this Wnt signal may be a potential mechanism by which p120^ctn^ attenuates Kaiso-mediated regulation of its target genes promoter. Additionally, this may be a potential mechanism by which Wnt signaling promotes the association between β-catenin and TCF/LEF, alleviating Kaiso’s inhibition of this heterodimer formation, and thus activating Wnt target genes.

Chaudhary et al. (2013) has previously shown p120^ctn^ is localized to the nucleus in a Kaiso over-expressing transgenic mouse model that exhibited intestinal inflammation. Whether nuclear p120^ctn^ translocation is a cause or consequence of Kaiso-induced inflammation remains to be resolved. The implications of p120^ctn^'s interaction with the transcription factor Kaiso (van Hengel et al., 1999; Kelly et al., 2004; Reynolds and Roczniak-Ferguson, 2004; Daniel, 2007; McCrea and Gottardi, 2016) (Figure 3), will be further discussed below.

### The transcription factor Kaiso and intestinal barrier dysfunction

Over two decades ago, Kaiso was discovered as a binding partner for p120^ctn^ (Daniel and Reynolds, 1999; Kelly and Daniel, 2006). To gain insight into the role of p120^ctn^ in AJs, a yeast-two hybrid screen was conducted to identify p120^ctn^ binding partners. The most frequent gene identified encoded the novel uncharacterized protein that was named Kaiso (Daniel and Reynolds, 1999). Kaiso is a member of the Broad complex, Tramtrack and Bric à brac/Poxvirus and Zinc finger (BTB/POZ) transcription factor family, characterized by an amino-terminal BTB/POZ domain that facilitates protein-protein interactions and three zinc fingers at its carboxy-terminal that mediate DNA binding (Kelly and Daniel, 2006; Daniel, 2007; Pierre et al., 2019). Initial characterization showed that Kaiso binds to DNA *via* a specific sequence called the Kaiso binding site (KBS) or to methylated CpG dinucleotides (Daniel et al., 2002; Kelly and Daniel, 2006). It was later shown that Kaiso can also bind to a methylated TCTCGCGAGA motif (Raghav et al., 2012; Pierre et al., 2019). As a dual-specificity DNA-binding transcription factor, Kaiso functions as either a transcriptional activator or repressor (Daniel, 2007; Iioka et al., 2009; Pierre et al., 2019), which further adds to the complexity of its role in normal vertebrate development and disease states such as IBD and colon cancer. Notably, several Kaiso target genes are also canonical Wnt target genes (e.g., *Cyclin D1*, *c-Myc* and *matrilysin*) (Park et al., 2005; Spring et al., 2005; Pierre et al., 2019). Since canonical Wnt signaling is essential for normal intestinal homeostasis, studies from our lab and others have explored Kaiso’s role in the mammalian intestine.

Insights into Kaiso’s roles in the intestine were garnered primarily from transgenic and knock-out mouse models. In the context of the *Apc* ^*Min/+*^ mouse model of colon cancer, Prokhortchouk *et al.,* found that *Kaiso* ^*null*^ *; Apc* ^*Min/+*^ mice displayed delayed polyp growth and an increased lifespan (Prokhortchouk et al., 2006). This was an unexpected finding given that Kaiso repressed several Wnt target genes and Kaiso overexpression in the *Xenopus* model rescued the Wnt/β-catenin duplicate axis phenotype (Park et al., 2005; Donaldson et al., 2012; Jiang et al., 2012). To better understand these paradoxical findings, Chaudhary *et al.* generated an intestinal-specific Kaiso overexpressing mouse model (*Kaiso* ^*Tg*^) (Chaudhary et al., 2013). Surprisingly the *Kaiso* ^*Tg*^ mice exhibited morphological changes (e.g., villi blunting, villi fusion, crypt hyperplasia and expansion), intestinal inflammation characterized by neutrophilia, and increased intestinal permeability (Chaudhary et al., 2013; Robinson et al., 2019). When *Kaiso* ^*Tg*^ were mated with the *Apc* ^*Min/+*^ mouse model of colon cancer, the resulting *Kaiso* ^*Tg*^ *;Apc* ^*Min/+*^ progeny exhibited ∼3-fold more (but smaller) polyps and reduced life span (Pierre et al., 2015). Together, these findings coupled with the findings of Prokhortchouk *et al.* support a role for Kaiso in promoting intestinal tumorigenesis (Prokhortchouk et al., 2006; Pierre et al., 2015). This role is further underscored by the fact that *Apc* ^*Min/+*^ polyps, human colon cancer and Crohn’s disease tissues display high Kaiso levels relative to normal tissues (Pierre et al., 2015; Robinson et al., 2019).

The contradictory findings observed from Kaiso overexpression and knock-out in different model systems may be due to the presence of the Kaiso-like transcription factor ZBTB4. Like Kaiso, ZBTB4 is a BTB/POZ protein that functions as a transcriptional repressor and like Kaiso, ZBTB4 also binds to DNA *via* both the KBS and methylated CpG sites (Filion et al., 2006). Thus, in the absence of Kaiso, ZBTB4 may compensate for some Kaiso functions and regulate the expression of essential Kaiso targets genes (Pierre et al., 2019). However, ZBTB4 does not heterodimerize with Kaiso and it does not interact with p120^ctn^ (Filion et al., 2006). Interestingly, in contrast to Kaiso that is highly expressed in many aggressive cancers (Pierre et al., 2019), ZBTB4 is expressed at low levels in several cancers including colorectal cancer (Weber et al., 2008; Xiang et al., 2020) and is postulated to be a tumor suppressor since it inhibits cell proliferation and induces cell cycle arrest and apoptosis in Ewing sarcoma (Yu et al., 2018). As there have been few studies examining the function of ZBTB4 in the mammalian intestine or canonical Wnt signaling, further studies are needed to determine if ZBTB4 does in fact function somewhat redundantly in the absence of Kaiso and its potential roles in intestinal diseases.

### Kaiso’s interaction with AJ proteins and the implications for intestinal pathologies

Several studies have shown that Kaiso directly or indirectly regulates the expression of various proteins in apical junctional complexes (e.g., ZO-1, E-cadherin) (Jones et al., 2012; Bassey-Archibong et al., 2016; Pierre et al., 2019; Robinson et al., 2019), which has important implications for diseases such as IBD and colon cancer (Pierre et al., 2015; Robinson et al., 2019). Since Kaiso primarily localized to the nucleus (Daniel and Reynolds, 1999), Kaiso’s identification as a p120^ctn^ binding partner was the first hint that p120^ctn^, like β-catenin, could translocate to the nucleus (Kelly et al., 2004; Daniel, 2007). Studies have since revealed that Kaiso binds preferentially to p120^ctn^ isoform-3A compared to p120^ctn^ isoform-1A in colorectal cancer and lung cancer cells (Daniel and Reynolds, 1999; Liu et al., 2014). p120^ctn^ uses Arm repeats 1-7 to interact with a carboxy-terminal region flanking Kaiso’s DNA-binding zinc finger domain (Daniel and Reynolds, 1999). Consequently, nuclear p120^ctn^ prevents Kaiso’s DNA-binding and inhibits Kaiso’s transcriptional activity and regulation of Wnt target genes such as *Siamois* and *matrilysin* (Daniel et al., 2002; Park et al., 2005; Spring et al., 2005; Daniel, 2007) (Figure 3 and Table 1).

Loss of p120^ctn^ at the plasma membrane results in defective AJ structure and integrity, and contributes to intestinal inflammation in mice (Smalley-Freed et al., 2010). In the inflamed intestines of *Kaiso* ^*Tg*^ mice, p120^ctn^ exhibits reduced membrane localization and increased nuclear localization, where it presumably interacts with Kaiso (Chaudhary et al., 2013). Importantly, p120^ctn^ also utilizes Arm repeats 1-7 to bind to E-cadherin, suggesting that p120^ctn^’s association with Kaiso and E-cadherin is mutually exclusive (Daniel and Reynolds, 1995; Daniel and Reynolds, 1999; Daniel et al., 2002). This would affect E-cadherin stability and turnover at the AJ, rendering the integrity of the AJ non-functional. Robinson *et al.* found that E-cadherin protein expression and membrane localization were decreased in the *Kaiso* ^*Tg*^ mice (Robinson et al., 2019). As such, the change in p120^ctn^’s subcellular localization and concomitant decrease in E-cadherin expression hints at one possible mechanism by which Kaiso disrupts intestinal barrier integrity in *Kaiso* ^*Tg*^ mice (Chaudhary et al., 2013; Robinson et al., 2019). Importantly, however, Kaiso also binds to and represses the *CDH1* gene (which encodes E-cadherin) in a methylation-dependent manner (Jones et al., 2012; Jones et al., 2014). Kaiso-depletion and demethylation treatment with 5-aza-2′-deoxycytidine in prostate and breast tumour cell lines resulted in a substantial increase in E-cadherin mRNA expression (Jones et al., 2012; Jones et al., 2014). The repressive impact of Kaiso on *CDH1* gene expression offers another mechanism by which Kaiso overexpression may potentiate intestinal inflammation and permeability in *Kaiso* ^*Tg*^ mice. Whether the intestinal inflammation observed in *Kaiso* ^*Tg*^ mice is the result of nuclear p120^ctn^ localization, *CDH1* transcriptional repression, or both, warrants further investigation and is one current focus of our laboratory.

In addition to p120^ctn^, one study revealed that Kaiso also associates with β-catenin (del Valle-Pérez et al., 2016). β-catenin interacts with Kaiso using Arm repeats 1-6, which partially overlaps with the region required for binding TCF (Arm repeats 3–10). In fact, ectopic Kaiso expression has been found to abrogate binding between β-catenin and TCF (Park et al., 2005; del Valle-Pérez et al., 2016), while Kaiso depletion enhanced this interaction (Park et al., 2005). Furthermore, Liu et al. (2014) demonstrated that Kaiso binds to CpG dinucleotides in the *CTNNB1* gene promoter (which encodes β-catenin) in a methylation-dependent manner and that high Kaiso expression repressed transcription of β-catenin mRNA. As such, Kaiso directly impacts canonical Wnt signaling at the DNA level (by repressing gene expression of canonical Wnt target genes and *CTNNB1*), and protein level (by binding to nuclear β-catenin) (Park et al., 2005; Liu et al., 2014). Importantly, p120^ctn^ has been shown to interfere with Kaiso’s repression of the β-catenin/TCF4 transcription complex in SW480 colon cancer cells (del Valle-Pérez et al., 2016). del Valle-Pérez et al. (2016) showed that Wnt stimulation resulted in p120^ctn^ nuclear translocation that disrupted Kaiso-mediated transcriptional repression and potentiated Wnt/β-catenin signaling. The antagonistic relationship between Kaiso and p120^ctn^ suggests that these two proteins may have opposing roles in regulating the Wnt/β-catenin pathway in intestinal tissues (Figure 3). These observations may explain the increased tumour burden, decreased lifespan, and increased Wnt target gene expression in *Kaiso* ^*Tg*^ *;Apc* ^*Min/+*^ mice (Pierre et al., 2015), although this is yet to be tested empirically.

Collectively, these findings demonstrate that several key AJ proteins interact with Kaiso in the nucleus of intestinal epithelial cells. However, more studies are necessary to fully elucidate how (or whether) Kaiso’s interaction with p120^ctn^, β-catenin and E-cadherin contribute to the neutrophil-driven inflammation in *Kaiso* ^*Tg*^ murine intestines, and the implications this has for diseases like IBD and CRC. Future studies also need to consider the role of ZBTB4 in Kaiso-mediated regulation of gene expression and biological processes such as inflammation, cell proliferation, apoptosis and cell motility.

## Concluding remarks

AJ proteins have emerged as key players in the regulation of cellular signaling in addition to their classical structural functions at the epithelial cell membrane. Imbalances in the structural and signaling properties of AJ proteins can result in the development of intestinal diseases. Given the fundamental role that nuclear β-catenin plays in canonical Wnt signaling and the essential role Wnt plays in intestinal homeostasis, the biological functions of nuclear β-catenin in the intestine are well established. However, as highlighted above, additional investigation into the nuclear roles of other AJ proteins such as p120^ctn^ is deserving of further attention to address some outstanding questions. For example, what are the cue(s) that signal the nuclear translocation of AJ proteins? Is nuclear translocation of AJ proteins a cause or consequence of loss of epithelial barrier integrity? Are all AJ proteins capable of binding to DNA and regulating transcription?

Additionally, several studies have shown that the transcription factor Kaiso plays a role in intestinal inflammation and tumorigenesis in mice, and that these pathologies may be influenced by Kaiso’s regulation of E-cadherin and nuclear interactions with p120^ctn^ and β-catenin. However, the upstream signals that dictate Kaiso’s interaction with AJ proteins, and whether Kaiso interacts with other members of the AJ (e.g., α-and γ-catenin) or other proteins in the apical junctional complex (e.g., ZO-1, claudins) are currently unknown. Furthermore, the possibility of a redundant function between Kaiso and ZBTB4 warrants further investigation to fully understand the role of Kaiso in faulty cell adhesion, Wnt signaling, and intestinal disease.

Studies linking mislocalization of AJ proteins, ectopic Kaiso overexpression, and nuclear interactions between Kaiso and AJ proteins, hint at a possible mechanism by which Kaiso (and possibly other transcription factors) potentiates intestinal inflammation in mammalian tissues. These interactions have vast implications for disease processes where intestinal epithelial integrity is an important and essential factor to maintain intestinal homeostasis. Additional studies utilizing *in vivo* mouse models will be vital for elucidating and shedding much needed light on the cellular contexts involved in these interactions.

## References

[B1] Aktary Z. Alaee M. Pasdar M. (2017). Beyond cell-cell adhesion: Plakoglobin and the regulation of tumorigenesis and metastasis. Oncotarget 8 (19), 32270–32291. 10.18632/oncotarget.15650 28416759 PMC5458283

[B2] Aktary Z. Kulak S. Mackey J. Jahroudi N. Pasdar M. (2013). Plakoglobin interacts with the transcription factor p53 and regulates the expression of 14-3-3σ. J. Cell Sci. 126 (14), 3031–3042. 10.1242/jcs.120642 23687381

[B3] Aktary Z. Pasdar M. (2012). Plakoglobin: Role in tumorigenesis and metastasis. Int. J. Cell Biol. 2012, 189521. 10.1155/2012/189521 22481945 PMC3312339

[B4] Ali A. Tan H. Y. Kaiko G. E. (2020). Role of the intestinal epithelium and its interaction with the microbiota in food allergy. Front. Immunol. 1–12. 10.3389/fimmu.2020.604054 PMC7750388 33365031

[B5] Anthony C. C. Robbins D. J. Ahmed Y. Lee E. (2020). Nuclear regulation of wnt/β-catenin signaling: It's a complex situation. Genes (Basel) 11 (8), 1–11. 10.3390/genes11080886 PMC7465203 32759724

[B6] Arnold A. Tronser M. Sers C. Ahadova A. Endris V. Mamlouk S. (2020). The majority of β-catenin mutations in colorectal cancer is homozygous. BMC Cancer 20 (1), 1038–1110. 10.1186/s12885-020-07537-2 33115416 PMC7594410

[B7] Barrett J. C. Barrett J. C. Lee C. W. Lees N. J. Prescott C. A. Anderson A. (2009). Genome-wide association study of ulcerative colitis identifies three new susceptibility loci, including the HNF4A region. Nat. Genet. 41 (12), 1330–1334. 10.1038/ng.483 19915572 PMC2812019

[B8] Bassey-Archibong B. I. Kwiecien J. M. Milosavljevic S. B. Hallett R. M. Rayner L. G. A. Erb M. J. (2016). Kaiso depletion attenuates transforming growth factor-β signaling and metastatic activity of triple-negative breast cancer cells. Oncogenesis 5 (3), e208. 10.1038/oncsis.2016.17 26999717 PMC4815049

[B9] Beckers C. M. L. García-Vallejo J. J. van Hinsbergh V. W. M. van Nieuw Amerongen G. P. (2008). Nuclear targeting of β-catenin and p120ctn during thrombin-induced endothelial barrier dysfunction. Cardiovasc Res. 79 (4), 679–688. 10.1093/cvr/cvn127 18490349

[B10] Behrens J. von Kries J. P. Kühl M. Bruhn L. Wedlich D. Grosschedl R. (1996). Functional interaction of β-catenin with the transcription factor LEF-1. Nature 382 (6592), 638–642. 10.1038/382638a0 8757136

[B11] Benjamin J. M. Nelson W. J. (2008). Bench to bedside and back again: Molecular mechanisms of α-catenin function and roles in tumorigenesis. Seminars Cancer Biol. 18 (1), 53–64. 10.1016/j.semcancer.2007.08.003 PMC2692220 17945508

[B12] Bienz M. (2004). β-Catenin: A pivot between cell adhesion and Wnt signaling. Curr. Biol. 15, 64–67. 10.1016/j.cub.2004.12.058 15668160

[B13] Bondow B. J. Faber M. L. Wojta K. J. Walker E. M. Battle M. A. (2012). E-cadherin is required for intestinal morphogenesis in the mouse. Dev. Biol. 371 (1), 1–12. 10.1016/j.ydbio.2012.06.005 22766025 PMC3455111

[B14] Bujko M. Kober P. Mikula M. Ligaj M. Ostrowski O. Siedlecki J. A. (2015). Expression changes of cell-cell adhesion-related genes in colorectal tumors. Oncol. Lett. 9, 2463–2470. 10.3892/ol.2015.3107 26137091 PMC4473523

[B15] Chaudhary R. Pierre C. C. Nanan K. Wojtal D. Morone S. Pinelli C. (2013). The POZ-ZF transcription factor kaiso (ZBTB33) induces inflammation and progenitor cell differentiation in the murine intestine. PLoS One 8 (9), e74160. 10.1371/journal.pone.0074160 24040197 PMC3764064

[B16] Chen S. Liu J. Li G. Mo F. Xu X. Zhang T. (2008). Altered distribution of β-catenin and prognostic roles in colorectal carcinogenesis. Scand. J. Gastroenterology 43 (4), 456–464. 10.1080/00365520701785194 18365911

[B17] Chen Z. He X. Jia M. Liu Y. Qu D. Wu D. (2013). β-Catenin overexpression in the nucleus predicts progress disease and unfavourable survival in colorectal cancer: A meta-analysis. PLoS One 8 (5), 1–9. 10.1371/journal.pone.0063854 PMC3663842 23717499

[B161] Cheng X. Xu X. Chen D. Zhao F. Wang W. (2019). Therapeutic potential of targeting the Wnt/β-catenin signaling pathway in colorectal cancer. Biomed. Pharmacother. 110, 473–481. 30530050 10.1016/j.biopha.2018.11.082

[B18] Cheng G. Yang S. Zhang G. Xu Y. Liu X. Sun W. (2016). Lipopolysaccharide-induced &alpha;-catenin downregulation enhances the motility of human colorectal cancer cells in an NF-&kappa;B signaling-dependent manner. Ott 9, 7563–7571. 10.2147/ott.s123986 PMC5167382 28008274

[B19] Chitaev N. A. Averbakh A. Z. Troyanovsky R. B. Troyanovsky S. M. (1998). Molecular organization of the desmoglein-plakoglobin complex. J. Cell Sci. 111 (14), 1941–1949. 10.1242/jcs.111.14.1941 9645942

[B20] Choi S. H. Estarás C. Moresco J. J. Yates J. R. Jones K. A. (2013). α-Catenin interacts with APC to regulate β-catenin proteolysis and transcriptional repression of Wnt target genes. Genes Dev. 27 (22), 2473–2488. 10.1101/gad.229062.113 24240237 PMC3841736

[B21] Cong F. Schweizer L. Chamorro M. Varmus H. (2003). Requirement for a nuclear function of β-catenin in Wnt signaling. Mol. Cell Biol. 23 (23), 8462–8470. 10.1128/mcb.23.23.8462-8470.2003 14612392 PMC262677

[B163] Daniels D. L. Weis W. I. (2005). β-catenin directly displaces Groucho/TLE repressors from Tcf/Lef in Wnt-mediated transcription activation. Nat. Struct. Mol. Biol. 12 (4), 364–371. 15768032 10.1038/nsmb912

[B22] Daniel J. M. (2007). Dancing in and out of the nucleus: p120ctn and the transcription factor kaiso. Biochimica Biophysica Acta (BBA) - Mol. Cell Res. 1773 (1), 59–68. 10.1016/j.bbamcr.2006.08.052 17050009

[B23] Daniel J. M. Reynolds A. B. (1999). The catenin p120 ctn interacts with kaiso, a novel BTB/POZ domain zinc finger transcription factor. Mol. Cell Biol. 19 (5), 3614–3623. 10.1128/mcb.19.5.3614 10207085 PMC84161

[B24] Daniel J. M. Reynolds A. B. (1995). The tyrosine kinase substrate p120cas binds directly to E-cadherin but not to the adenomatous polyposis coli protein or alpha-catenin. Mol. Cell Biol. 15 (9), 4819–4824. 10.1128/mcb.15.9.4819 7651399 PMC230726

[B25] Daniel J. M. Spring C. M. Crawford H. C. Reynolds A. B. Baig A. (2002). The p120ctn-binding partner Kaiso is a bi-modal DNA-binding protein that recognizes both a sequence-specific consensus and methylated CpG dinucleotides. Nucleic Acids Res. 30 (13), 2911–2919. 10.1093/nar/gkf398 12087177 PMC117053

[B26] Daugherty R. L. Serebryannyy L. Yemelyanov A. Flozak A. S. Yu H. J. Kosak S. T. (2014). α-Catenin is an inhibitor of transcription. Proc. Natl. Acad. Sci. U.S.A. 111 (14), 5260–5265. 10.1073/pnas.1308663111 24706864 PMC3986139

[B27] Davis M. A. Reynolds A. B. (2006). Blocked acinar development, E-cadherin reduction, and intraepithelial neoplasia upon ablation of p120-catenin in the mouse salivary gland. Dev. Cell 10 (1), 21–31. 10.1016/j.devcel.2005.12.004 16399075

[B28] del Valle-Pérez B. Casagolda D. Lugilde E. Valls G. Codina M. Dave N. (2016). Wnt controls the transcriptional activity of Kaiso through CK1ε-dependent phosphorylation of p120-catenin. J. Cell Sci. 129 (4), 873. 10.1242/jcs.186288 26905970

[B29] Donaldson N. S. Pierre C. C. Anstey M. I. Robinson S. C. Weerawardane S. M. Daniel J. M. (2012). Kaiso represses the cell cycle gene cyclin D1 *via* sequence-specific and methyl-CpG-dependent mechanisms. PLoS One 7 (11), e50398. 10.1371/journal.pone.0050398 23226276 PMC3511522

[B30] El-Bahrawy M. Talbot I. Poulsom R. Alison M. (2002). Variable nuclear localization of α-catenin in colorectal carcinoma. Lab. Invest. 82 (9), 1167–1174. 10.1097/01.lab.0000028821.41246.6a 12218077

[B31] Fagotto F. Glück U. Gumbiner B. M. (1998). Nuclear localization signal-independent and importin/karyopherin-independent nuclear import of β-catenin. Curr. Biol. 8 (4), 181–190. 10.1016/s0960-9822(98)70082-x 9501980

[B32] Ferber E. C. Kajita M. Wadlow A. Tobiansky L. Niessen C. Ariga H. (2008). A role for the cleaved cytoplasmic domain of E-cadherin in the nucleus. J. Biol. Chem. 283 (19), 12691–12700. 10.1074/jbc.M708887200 18356166 PMC2442316

[B33] Fevr T. Robine S. Louvard D. Huelsken J. (2007). Wnt/β-Catenin is essential for intestinal homeostasis and maintenance of intestinal stem cells. Mol. Cell Biol. 27 (21), 7551–7559. 10.1128/mcb.01034-07 17785439 PMC2169070

[B34] Filion G. J. P. Zhenilo S. Salozhin S. Yamada D. Prokhortchouk E. Defossez P-A. (2006). A family of human zinc finger proteins that bind methylated DNA and repress transcription. Mol. Cell Biol. 26 (1), 169–181. 10.1128/mcb.26.1.169-181.2006 16354688 PMC1317629

[B35] Garcia-Gras E. Lombardi R. Giocondo M. J. Willerson J. T. Schneider M. D. Khoury D. S. (2006). Suppression of canonical Wnt/ -catenin signaling by nuclear plakoglobin recapitulates phenotype of arrhythmogenic right ventricular cardiomyopathy. J. Clin. Investigation 116 (7), 2012–2021. 10.1172/jci27751 PMC1483165 16823493

[B36] Giannini A. L. Vivanco M. D. M. Kypta R. M. (2000). α-Catenin inhibits β-catenin signaling by preventing formation of a β-Catenin·T-cell factor·DNA complex. J. Biol. Chem. 275 (29), 21883–21888. 10.1074/jbc.m001929200 10896949

[B37] Guo Z. Neilson L. J. Zhong H. Murray P. S. Zanivan S. Zaidel-Bar R. (2014). E-cadherin interactome complexity and robustness resolved by quantitative proteomics. Sci. Signal 7 (354), rs7–13. 10.1126/scisignal.2005473 25468996 PMC4972397

[B38] Hankey W. Frankel W. L. Groden J. (2018). Functions of the APC tumor suppressor protein dependent and independent of canonical WNT signaling: Implications for therapeutic targeting. Cancer Metastasis Rev. 37 (1), 159–172. 10.1007/s10555-017-9725-6 29318445 PMC5803335

[B39] Hao X. P. Pretlow T. G. Rao J. S. Pretlow T. P. (2001). β-Catenin expression is altered in human colonic aberrant crypt foci. Cancer Res. 216, 8085–8088. 11719432

[B40] Hartsock A. Nelson W. J. (2008). Adherens and tight junctions: Structure, function and connections to the Actin cytoskeleton. Biochimica Biophysica Acta (BBA) - Biomembr. 1778 (3), 660–669. 10.1016/j.bbamem.2007.07.012 PMC2682436 17854762

[B41] Henderson B. R. (2000). Nuclear-cytoplasmic shuttling of APC regulates β-catenin subcellular localization and turnover. Nat. Cell Biol. 2 (9), 653–660. 10.1038/35023605 10980707

[B42] Hermiston M. L. Gordon J. I. (1995). *In vivo* analysis of cadherin function in the mouse intestinal epithelium: Essential roles in adhesion, maintenance of differentiation, and regulation of programmed cell death. J. Cell Biol. 129 (2), 489–506. 10.1083/jcb.129.2.489 7721948 PMC2199905

[B159] Herrenknecht K. Ozawa M. Eckerskorn C. Lottspeich F. Lenter M. Kemler R. (1991). The uvomorulin-anchorage protein α catenin is a vinculin homologue. Proc. Natl. Acad. Sci. U. S. A. 88 (20), 9156–9160. 1924379 10.1073/pnas.88.20.9156 PMC52671

[B43] Hollander D. (1999). Intestinal permeability, leaky gut, and intestinal disorders. Curr. Gastroenterol. Rep. 1 (5), 410–416. 10.1007/s11894-999-0023-5 10980980

[B44] Hu G. (2012). p120-catenin: A novel regulator of innate immunity and inflammation. Crit. Rev. Immunol. 32 (2), 127–138. 10.1615/critrevimmunol.v32.i2.20 23216611 PMC3533242

[B45] Hulpiau P. Gul I. S. van Roy F. (2013). New insights into the evolution of metazoan cadherins and catenins. Prog. Mol. Biol. Transl. Sci. 116, 71–94. 10.1016/b978-0-12-394311-8.00004-2 23481191

[B46] Iioka H. Doerner S. K. Tamai K. (2009). Kaiso is a bimodal modulator for Wnt/β-catenin signaling. FEBS Lett. [Internet] 583 (4), 627–632. 10.1016/j.febslet.2009.01.012 19166851

[B47] Ikeda S. Kishida S. Yamamoto H. Murai H. Koyama S. Kikuchi A. (1998). Axin, a negative regulator of the Wnt signaling pathway, forms a complex with GSK-3beta and beta -catenin and promotes GSK-3beta -dependent phosphorylation of beta -catenin. EMBO J. 17 (5), 1371–1384. 10.1093/emboj/17.5.1371 9482734 PMC1170485

[B48] Ikeda W. Nakanishi H. Miyoshi J. Mandai K. Ishizaki H. Tanaka M. (1999). Afadin. J. Cell Biol. 146 (5), 1117–1132. 10.1083/jcb.146.5.1117 10477764 PMC2169488

[B49] Ireton R. C. Davis M. A. van Hengel J. Mariner D. J. Barnes K. Thoreson M. A. (2002). A novel role for p120 catenin in E-cadherin function. J. Cell Biol. 159 (3), 465–476. 10.1083/jcb.200205115 12427869 PMC2173073

[B50] Jiang G. Wang Y. Dai S. Liu Y. Stoecker M. Wang E. (2012). P120-catenin isoforms 1 and 3 regulate proliferation and cell cycle of lung cancer cells *via* β-catenin and Kaiso respectively. PLoS One 7 (1), e30303. 10.1371/journal.pone.0030303 22276175 PMC3262806

[B51] Johnson V. Volikos E. Halford S. E. Sadat E. T. E. Popat S. Talbot I. (2005). Exon 3 -catenin mutations are specifically associated with colorectal carcinomas in hereditary non-polyposis colorectal cancer syndrome. Gut 54 (2), 264–267. 10.1136/gut.2004.048132 15647192 PMC1774848

[B52] Jones J. Wang H. Karanam B. Theodore S. Dean-Colomb W. Welch D. R. (2014). Nuclear localization of Kaiso promotes the poorly differentiated phenotype and EMT in infiltrating ductal carcinomas. Clin. Exp. Metastasis 31 (5), 497–510. 10.1007/s10585-014-9644-7 24570268 PMC4065802

[B53] Jones J. Wang H. Zhou J. Hardy S. Turner T. Austin D. (2012). Nuclear kaiso indicates aggressive prostate cancers and promotes migration and invasiveness of prostate cancer cells. Am. J. Pathology 181 (5), 1836–1846. 10.1016/j.ajpath.2012.08.008 PMC3483816 22974583

[B162] Kam Y. Quaranta V. (2009). Cadherin-bound β-catenin feeds into the Wnt pathway upon adherens junctions dissociation: Evidence for an intersection between β-catenin pools. PLoS One. 4 (2). 10.1371/journal.pone.0004580 PMC2640460 19238201

[B54] Kameyama H. Nagahashi T. Shimada T. Tajima Y. Ichikawa K. Nakano J. (2018). Genomic characterization of colitis-associated colorectal cancer. World J. Surg. Oncol. 16, 121–129. 10.1186/s12957-018-1428-0 29966533 PMC6027567

[B55] Kelly K. F. Daniel J. M. (2006). POZ for effect - POZ-ZF transcription factors in cancer and development. Trends Cell Biol. 16 (11), 578–587. 10.1016/j.tcb.2006.09.003 16996269

[B56] Kelly K. F. Spring C. M. Otchere A. A. Daniel J. M. (2004). NLS-dependent nuclear localization of p120ctnis necessary to relieve Kaiso-mediated transcriptional repression. J. Cell Sci. 117 (13), 2675–2686. 10.1242/jcs.01101 15138284

[B57] Kim W. K. Kwon Y. Jang M. Park M. Kim J. Cho S. (2019). β-catenin activation down-regulates cell-cell junction-related genes and induces epithelial-to-mesenchymal transition in colorectal cancers. Sci. Rep. 9 (1), 1–15. 10.1038/s41598-019-54890-9 31804558 PMC6895046

[B58] Kitaeva M. N. Grogan L. Williams J. P. Dimond E. Nakahara K. Hausner P. (1997). Mutations in beta-catenin are uncommon in colorectal cancer occurring in occasional replication error-positive tumors. Cancer Res. 57 (20), 4478–4481. 9377556

[B164] Kolligs F. T. Hu G. Dang C. V. Fearon E. R. (1999). Neoplastic Transformation of RK3E by Mutant β-Catenin Requires Deregulation of Tcf/Lef Transcription but Not Activation of c- myc Expression. Mol. Cell. Biol. 19 (8), 5696–5706. 10409758 10.1128/mcb.19.8.5696 PMC84421

[B59] Kolligs F. T. Kolligs B. Hajra K. M. Hu H. Tani M. Cho R. C. (2000). γ-Catenin is regulated by the APC tumor suppressor and its oncogenic activity is distinct from that of β-catenin. Genes Dev. 14 (11), 1319–1331. 10.1101/gad.14.11.1319 10837025 PMC316666

[B60] Landy J. Ronde E. English N. Clark S. K. Hart A. L. Knight S. C. (2016). Tight junctions in inflammatory bowel diseases and inflammatory bowel disease associated colorectal cancer. Wjg 22 (11), 3117–3126. 10.3748/wjg.v22.i11.3117 27003989 PMC4789987

[B61] Lechuga S. Ivanov A. I. (2017). Disruption of the epithelial barrier during intestinal inflammation: Quest for new molecules and mechanisms. Biochimica Biophysica Acta (BBA) - Mol. Cell Res. 1864 (7), 1183–1194. 10.1016/j.bbamcr.2017.03.007 PMC5507344 28322932

[B62] Lee M. Ji H. Furuta Y. Park J. I. McCrea P. D. (2014). p120-catenin regulates REST and CoREST, and modulates mouse embryonic stem cell differentiation. J. Cell Sci. 127 (18), 4037–4051. 10.1242/jcs.151944 25074806 PMC4163646

[B158] Lewis J. S. Wahl J. K. Sass K. M. Jensen P. J. Johnson K. R. Wheelock M. J. (1997). Cross-talk between adherens junctions and desmosomes depends on plakoglobin. J. Cell Biol. 136 (4), 919–934. 9049256 10.1083/jcb.136.4.919 PMC2132504

[B63] Lifschitz-Mercer B. Amitai R. Maymon B. B. S. Shechtman L. Czernobilsky B. Leider-Trejo L. (2001). Nuclear localization of β-catenin and plakoglobin in primary and metastatic human colonic carcinomas, colonic adenomas, and normal colon. Int. J. Surg. Pathol. 9 (4), 273–279. 10.1177/106689690100900403 12574842

[B64] Liu Y. Dong Q. Z. Wang S. Xu H. T. Miao Y. Wang L. (2014). Kaiso interacts with p120-catenin to regulate β-catenin expression at the transcriptional level. PLoS One 9 (2), e87537–9. 10.1371/journal.pone.0087537 24498333 PMC3911973

[B65] Lombardi R. da Graca Cabreira-Hansen M. Bell A. Fromm R. R. Willerson J. T. Marian A. J. (2011). Nuclear plakoglobin is essential for differentiation of cardiac progenitor cells to adipocytes in arrhythmogenic right ventricular cardiomyopathy. Circ. Res. 109 (12), 1342–1353. 10.1161/circresaha.111.255075 22021931 PMC3237769

[B66] Maeda O. Usami N. Kondo M. Takahashi M. Goto H. Shimokata K. (2004). Plakoglobin (γ-catenin) has TCF/LEF family-dependent transcriptional activity in β-catenin-deficient cell line. Oncogene 23 (4), 964–972. 10.1038/sj.onc.1207254 14661054

[B67] Mah A. T. Yan K. S. Kuo C. J. (2016). Wnt pathway regulation of intestinal stem cells. J. Physiol. 594 (17), 4837–4847. 10.1113/jp271754 27581568 PMC5009769

[B68] Maiden S. L. Hardin J. (2011). The secret life of α-catenin: Moonlighting in morphogenesis. J. Cell Biol. 195 (4), 543–552. 10.1083/jcb.201103106 22084304 PMC3257527

[B69] Maître J. L. Heisenberg C. P. (2013). Three functions of cadherins in cell adhesion. Curr. Biol. 23 (14), 626–633. 23885883 10.1016/j.cub.2013.06.019 PMC3722483

[B70] Mårtensson A. Öberg A. Jung A. Cederquist K. Stenling R. Palmqvist R. (2007). Β-Catenin expression in relation to genetic instability and prognosis in colorectal cancer. Oncol. Rep. 17 (2), 447–452. 17203186

[B71] Mayerle J. Friess H. Büchler M. W. Schnekenburger J. Weiss F. U. Zimmer K. P. (2003). Up-regulation, nuclear import, and tumor growth stimulation of the adhesion protein p120ctn in pancreatic cancer. Gastroenterology 124 (4), 949–960. 10.1053/gast.2003.50142 12671892

[B72] Mbom B. C. Nelson W. J. Barth A. (2013). β-catenin at the centrosome. BioEssays 35 (9), 804–809. 10.1002/bies.201300045 23804296 PMC3983869

[B73] McCrea P. D. Gottardi C. J. (2016). Beyond β-catenin: Prospects for a larger catenin network in the nucleus. Nat. Rev. Mol. Cell Biol. 17 (1), 55–64. 10.1038/nrm.2015.3 26580716 PMC4845654

[B74] Mehta S. Nijhuis A. Kumagai T. Lindsay J. Silver A. (2015). Defects in the adherens junction complex (E-cadherin/ β-catenin) in inflammatory bowel disease. Cell Tissue Res. 360 (3), 749–760. 10.1007/s00441-014-1994-6 25238996

[B75] Meng W. Takeichi M. (2009). Adherens junction: Molecular architecture and regulation. Cold Spring Harb. Perspect. Biol. 1, a002899. 10.1101/cshperspect.a002899 20457565 PMC2882120

[B76] Michielan A. D'Incà R. (2015). Intestinal permeability in inflammatory bowel disease: Pathogenesis, clinical evaluation, and therapy of leaky gut. Mediat. Inflamm. 2015, 628157. 10.1155/2015/628157 PMC4637104 26582965

[B77] Miravet S. Piedra J. Miró F. Itarte E. Garcıa de Herreros A. G. Duñach M. (2002). The transcriptional factor tcf-4 contains different binding sites for β-catenin and plakoglobin. J. Biol. Chem. 277 (3), 1884–1891. 10.1074/jbc.m110248200 11711551

[B78] Mis M. O'Brien S. Steinhart Z. Lin S. Hart T. Moffat J. (2020). IPO11 mediates βcatenin nuclear import in a subset of colorectal cancers. J. Cell Biol. 219 (2), 1–13. 10.1083/jcb.201903017 PMC7041691 31881079

[B79] Morgan R. G. Ridsdale J. Tonks A. Darley R. L. (2014). Factors affecting the nuclear localization of β-catenin in normal and malignant tissue. J. Cell. Biochem. 115 (8), 1351–1361. 10.1002/jcb.24803 24610469

[B80] Munemitsu S. Albert I. Souza B. Rubinfeld D. Polakis P. (1995). Regulation of intracellular beta-catenin levels by the adenomatous polyposis coli (APC) tumor-suppressor protein. Proc. Natl. Acad. Sci. U.S.A. 92 (7), 3046–3050. 10.1073/pnas.92.7.3046 7708772 PMC42356

[B81] Nagel J. M. Kriegl L. Horst D. Engel J. Gautam S. Mantzoros C. S. (2010). γ-Catenin is an independent prognostic marker in early stage colorectal cancer. Int. J. Colorectal Dis. 25 (11), 1301–1309. 10.1007/s00384-010-1046-y 20737155

[B82] Nagel J. M. Lahm H. Ofner A. Göke B. Kolligs F. T. (2017). γ-Catenin acts as a tumor suppressor through context-dependent mechanisms in colorectal cancer. Int. J. Colorectal Dis. 32 (9), 1243–1251. 10.1007/s00384-017-2846-0 28681073

[B83] Naydenov N. G. Lechuga S. Zalavadia A. Mukherjee P. K. Gordon I. O. Skvasik D. (2022). P-cadherin regulates intestinal epithelial cell migration and mucosal repair, but is dispensable for colitis associated colon cancer. Cells 11 (9). 10.3390/cells11091467 PMC9100778 35563773

[B84] Okumura N. Kagami T. Fujii K. Nakahara M. Koizumi N. (2018). Involvement of nectin-afadin in the adherens junctions of the corneal endothelium. Cornea 37 (5), 633–640. 10.1097/ico.0000000000001526 29384809

[B85] Orsulic S. Huber O. Aberle H. Arnold S. Kemler R. (1999). E-cadherin binding prevents beta-catenin nuclear localization and beta-catenin/LEF-1-mediated transactivation. J. Cell Sci. 112 (8), 1237–1245. 10.1242/jcs.112.8.1237 10085258

[B86] Otey C. A. Carpen O. (2004). ?-actinin revisited: A fresh look at an old player. Cell Motil. Cytoskelet. 58, 104–111. 10.1002/cm.20007 15083532

[B87] Pan H. Gao F. Papageorgis P. Abdolmaleky H. M. Faller D. V. Thiagalingam S. (2007). Aberrant activation of γ-catenin promotes genomic instability and oncogenic effects during tumor progression. Cancer Biol. Ther. 6 (10), 1638–1643. 10.4161/cbt.6.10.4904 18245958

[B88] Park J. I. Kim S. W. Lyons J. P. Ji H. Nguyen T. T. Cho K. (2005). Kaiso/p120-Catenin and TCF/β-Catenin complexes coordinately regulate canonical Wnt gene targets. Dev. Cell 8 (6), 843–854. 10.1016/j.devcel.2005.04.010 15935774

[B89] Peng X. Cuff L. E. Lawton C. D. DeMali K. A. (2010). Vinculin regulates cell-surface E-cadherin expression by binding to β-catenin. J. Cell Sci. 123 (4), 567–577. 10.1242/jcs.056432 20086044 PMC2818194

[B90] Peng X. Nelson E. S. Maiers J. L. DeMali K. A. (2011). New insights into vinculin function and regulation. Chem. Res. Toxicol. 28, 191–231. 10.1016/b978-0-12-386043-9.00005-0 PMC4426885 21414589

[B91] Perez-Moreno M. Davis M. A. Wong E. Pasolli H. A. Reynolds A. B. Fuchs E. (2006). P120-Catenin mediates inflammatory responses in the skin. Cell 124 (3), 631–644. 10.1016/j.cell.2005.11.043 16469707 PMC2443688

[B92] Perez-Moreno M. Song W. Pasolli H. A. Williams S. E. Fuchs E. (2008). Loss of p120 catenin and links to mitotic alterations, inflammation, and skin cancer. Proc. Natl. Acad. Sci. U.S.A. 105 (40), 15399–15404. 10.1073/pnas.0807301105 18809907 PMC2547465

[B93] Perochon J. Carroll L. R. Cordero J. B. (2018). Wnt signaling in intestinal stem cells: Lessons from mice and flies. Genes (Basel) 9 (3), 1–19. 10.3390/genes9030138 PMC5867859 29498662

[B94] Peterson L. W. Artis D. (2014). Intestinal epithelial cells: Regulators of barrier function and immune homeostasis. Nat. Rev. Immunol. 14 (3), 141–153. 10.1038/nri3608 24566914

[B95] Pierre C. C. Hercules S. M. Yates C. Daniel J. M. (2019). Dancing from bottoms up - roles of the POZ-ZF transcription factor kaiso in cancer. Biochimica Biophysica Acta (BBA) - Rev. Cancer 1871 (1), 64–74. [Internet]. 10.1016/j.bbcan.2018.10.005 PMC6467064 30419310

[B96] Pierre C. C. Longo J. Mavor M. Milosavljevic S. B. Chaudhary R. Gilbreath E. (2015). Kaiso overexpression promotes intestinal inflammation and potentiates intestinal tumorigenesis in ApcMin/+ mice. Biochimica Biophysica Acta (BBA) - Mol. Basis Dis. 1852 (9), 1846–1855. 10.1016/j.bbadis.2015.06.011 26073433

[B97] Prokhortchouk A. Sansom O. Selfridge J. Caballero I. M. Salozhin S. Aithozhina D. (2006). Kaiso-deficient mice show resistance to intestinal cancer. Mol. Cell Biol. 26 (1), 199–208. 10.1128/mcb.26.1.199-208.2006 16354691 PMC1317619

[B98] Raftopoulos I. Davaris P. Karatzas G. Karayannacos P. Kouraklis G. (1998). Level of α-catenin expression in colorectal cancer correlates with invasiveness, metastatic potential, and survival. J. Surg. Oncol. 68 (2), 92–99. 10.1002/(sici)1096-9098(199806)68:2<92:aid-jso4>3.0.co;2-f 9624037

[B99] Raghav S. K. Waszak S. M. Krier I. Gubelmann C. Isakova A. Mikkelsen T. S. (2012). Integrative genomics identifies the corepressor SMRT as a gatekeeper of adipogenesis through the transcription factors C/EBPβ and KAISO. Mol. Cell 46 (3), 335–350. 10.1016/j.molcel.2012.03.017 22521691

[B100] Ramena Y. Ramena G. (2018). Cell-cell junctions and epithelial differentiation. J. Morphol. Anat. 2 (1), 111.

[B101] Raya-Sandino A. Luissint A. C. Kusters D. H. M. Narayanan V. Flemming S. Garcia-Hernandez V. (2021). Regulation of intestinal epithelial intercellular adhesion and barrier function by desmosomal cadherin desmocollin-2. MBoC 32 (8), 753–768. 10.1091/mbc.e20-12-0775 33596089 PMC8108520

[B102] Rees W. D. Tandun R. Yau E. Zachos N. C. Steiner T. S. (2020). Regenerative intestinal stem cells induced by acute and chronic injury: The saving grace of the epithelium? Front. Cell Dev. Biol. 8, 583919–584015. 10.3389/fcell.2020.583919 33282867 PMC7688923

[B103] Reynolds A. B. Roczniak-Ferguson A. (2004). Emerging roles for p120-catenin in cell adhesion and cancer. Oncogene 23 (487), 7947–7956. 10.1038/sj.onc.1208161 15489912

[B104] Robinson S. C. Chaudhary R. Jiménez-Saiz R. Rayner L. G. A. Bayer L. Jordana M. (2019). Kaiso-induced intestinal inflammation is preceded by diminished E-cadherin expression and intestinal integrity. PLoS One 14, e0217220–17. 10.1371/journal.pone.0217220 31199830 PMC6568390

[B105] Roczniak-Ferguson A. Reynolds A. B. (2003). Regulation of p120-catenin nucleocytoplasmic shuttling activity. J. Cell Sci. 116 (20), 4201–4212. 10.1242/jcs.00724 12953069

[B106] Rosin-Arbesfeld R. Cliffe A. Brabletz T. Bienz M. (2003). Nuclear export of the APC tumour suppressor controls β-catenin function in transcription. EMBO J. 22 (5), 1101–1113. 10.1093/emboj/cdg105 12606575 PMC150338

[B107] Rubin D. C. Shaker A. Levin M. S. (2012). Chronic intestinal inflammation: Inflammatory bowel disease and colitis-associated colon cancer. Front. Immunol. 3, 107–110. 10.3389/fimmu.2012.00107 22586430 PMC3347037

[B108] Samowitz W. S. Powers M. D. Spirio L. N. Nollet F. van Roy F. Slattery M. L. (1999). Beta-catenin mutations are more frequent in small colorectal adenomas than in larger adenomas and invasive carcinomas. Cancer Res. 59 (7), 1442–1444. 10197610

[B109] Sanders D. S. A. Perry I. Hardy R. Jankowski J. (2000). Aberrant P-cadherin expression is a feature of clonal expansion in the gastrointestinal tract associated with repair and neoplasia. J. Pathol. 190 (5), 526–530. 10.1002/(sici)1096-9896(200004)190:5<526:aid-path564>3.0.co;2-9 10727977

[B110] Schneider M. R. Dahlhoff M. Horst D. Hirschi B. Trülzsch K. Müller-Höcker J. (2010). A key role for E-cadherin in intestinal homeostasis and paneth cell maturation. PLoS One 5 (12), e14325. 10.1371/journal.pone.0014325 21179475 PMC3001873

[B111] Schneider M. R. Kolligs F. T. (2015). E-cadherin's role in development, tissue homeostasis and disease: Insights from mouse models. BioEssays 37 (3), 294–304. 10.1002/bies.201400141 25449798

[B112] Secondulfo M. de Magistris L. Fiandra R. Caserta L. Belletta M. Tartaglione M. T. (2001). Intestinal permeability in Crohn's disease patients and their first degree relatives. Dig. Liver Dis. 33 (8), 680–685. 10.1016/s1590-8658(01)80045-1 11785714

[B113] Serebryannyy L. A. Yemelyanov A. Gottardi C. J. de Lanerolle P. (2017). Nuclear α-catenin mediates the DNA damage response via β-catenin and nuclear Actin. J. Cell Sci. 130 (10), 1717–1729. 10.1242/jcs.199893 28348105 PMC5450192

[B114] Shang S. Hua F. Hu Z. W. (2017). The regulation of β-catenin activity and function in cancer: Therapeutic opportunities. Oncotarget 8 (20), 33972–33989. 10.18632/oncotarget.15687 28430641 PMC5464927

[B115] Sharma M. Jamieson C. Lui C. Henderson B. R. (2016). Distinct hydrophobic "patches" in the N- and C-tails of beta-catenin contribute to nuclear transport. Exp. Cell Res. 348 (2), 132–145. 10.1016/j.yexcr.2016.09.009 27658570

[B116] Sheng H. Shao J. Williams C. S. Pereira M. A. Taketo M. M. Oshima M. (1998). Nuclear translocation of beta-catenin in hereditary and carcinogen- induced intestinal adenomas. Carcinogenesis 19 (4), 543–549. 10.1093/carcin/19.4.543 9600336

[B117] Sjöblom B. Salmazo A. Djinović-Carugo K. (2008). α-Actinin structure and regulation. Cell Mol. Life Sci. 65 (17), 2688–2701. 18488141 10.1007/s00018-008-8080-8 PMC11131806

[B118] Smalley-Freed W. G. Efimov A. Burnett P. E. Short S. P. Davis M. A. Gumucio D. L. (2010). P120-Catenin is essential for maintenance of barrier function and intestinal homeostasis in mice. J. Clin. Invest. 120 (6), 1824–1835. 10.1172/jci41414 20484816 PMC2877948

[B119] Smalley-Freed W. G. Efimov A. Short S. P. Jia P. Zhao Z. Washington M. K. (2011). Adenoma formation following limited ablation of p120-catenin in the mouse intestine. PLoS One 6 (5), e19880. 10.1371/journal.pone.0019880 21611205 PMC3096651

[B120] Solanas G. Batlle E. (2011). Control of cell adhesion and compartmentalization in the intestinal epithelium. Exp. Cell Res. 317 (19), 2695–2701. 10.1016/j.yexcr.2011.07.019 21820431

[B121] Sparks A. B. Morin P. J. Vogelstein B. Kinzler K. W. (1998). Mutational analysis of the APC/beta-catenin/Tcf pathway in colorectal cancer. Cancer Res. 58 (6), 1130–1134. 9515795

[B122] Spindler V. Meir M. Vigh B. Flemming S. Hütz K. Germer C. T. (2015). Loss of desmoglein 2 contributes to the pathogenesis of crohn's disease. Inflamm. Bowel Dis. 21 (10), 2349–2359. 10.1097/MIB.0000000000000486 26115074

[B123] Spring C. M. Kelly K. F. O'Kelly I. Graham M. Crawford H. C. Daniel J. M. (2005). The catenin p120ctn inhibits Kaiso-mediated transcriptional repression of the β-catenin/TCF target gene matrilysin. Exp. Cell Res. 305 (2), 253–265. 10.1016/j.yexcr.2005.01.007 15817151

[B124] Stanczak A. Stec R. Bodnar L. Olszewski W. Cichowicz M. Kozlowski W. (2011). Prognostic significance of wnt-1, β-catenin and E-cadherin expression in advanced colorectal carcinoma. Pathol. Oncol. Res. 17 (4), 955–963. 10.1007/s12253-011-9409-4 21678109 PMC3185231

[B125] Su Y. J. Chang Y. W. Lin W. H. Liang C. L. Lee J. L. (2015). An aberrant nuclear localization of E-cadherin is a potent inhibitor of Wnt/β-catenin-elicited promotion of the cancer stem cell phenotype. Oncogenesis 4 (6), e157. 10.1038/oncsis.2015.17 26075748 PMC4491612

[B126] Su Y. Y. Fu C. Ishikawa S. Stella A. Kojima M. Shitoh K. (2008). APC is essential for targeting phosphorylated β-catenin to the scfβ-TrCP ubiquitin ligase. Mol. Cell 32 (5), 652–661. 10.1016/j.molcel.2008.10.023 19061640

[B127] Suh E. K. Gumbiner B. M. (2003). Translocation of β-catenin into the nucleus independent of interactions with FG-rich nucleoporins. Exp. Cell Res. 290 (2), 447–456. 10.1016/s0014-4827(03)00370-7 14568002

[B128] Sun Y. Zhang J. Ma L. (2014). α-catenin. Cell Cycle 13 (15), 2334–2339. 10.4161/cc.29765 25483184 PMC4128878

[B129] Tachibana K. Nakanishi H. Mandai K. Ozaki K. Ikeda W. Yamamoto Y. (2000). Two cell adhesion molecules, nectin and cadherin, interact through their cytoplasmic domain-associated proteins. J. Cell Biol. 150 (5), 1161–1176. 10.1083/jcb.150.5.1161 10974003 PMC2175253

[B130] Takai Y. Ikeda W. Ogita H. Rikitake Y. (2008). The immunoglobulin-like cell adhesion molecule nectin and its associated protein afadin. Annu. Rev. Cell Dev. Biol. 24, 309–342. 10.1146/annurev.cellbio.24.110707.175339 18593353

[B131] Takai Y. Irie K. Shimizu K. Sakisaka T. Ikeda W. (2006). Nectins and nectin-like molecules: Roles in cell adhesion, migration, and polarization. Cancer Sci. 94 (8), 655–667. 10.1111/j.1349-7006.2003.tb01499.x PMC11160195 12901789

[B132] Takai Y. Miyoshi J. Ikeda W. Ogita H. (2008). Nectins and nectin-like molecules: Roles in contact inhibition of cell movement and proliferation. Nat. Rev. Mol. Cell Biol. 9 (8), 603–615. 10.1038/nrm2457 18648374

[B133] Takai Y. Nakanishi H. (2003). Nectin and afadin: Novel organizers of intercellular junctions. J. Cell Sci. 116 (1), 17–27. 10.1242/jcs.00167 12456712

[B160] Tanaka H. Kawaguchi M. Shoda S. Miyoshi T. Iwasaki R. Hyodo F. (2019). Nuclear accumulation of β-catenin in cancer stem cell radioresistance and stemness in human colon cancer. Anticancer Res. 39 (12), 6575–6583. 31810923 10.21873/anticanres.13873

[B165] Tetsu O. McCormick F. (1999). Beta-Catenin regulates expression of cyclinD1 in colon carcinoma cells. Nature. 398, 422. 10201372 10.1038/18884

[B134] Tewari R. Bailes E. Bunting K. A. Coates J. C. (2010). Armadillo-repeat protein functions: Questions for little creatures. Trends Cell Biol. 20 (8), 470–481. 10.1016/j.tcb.2010.05.003 20688255

[B135] Tsanou E. Peschos D. Batistatou A. Charalabopoulos A. Charalabopoulos K. (2008). The E-cadherin adhesion molecule and colorectal cancer. A global literature approach. Anticancer Res. 28, 3815–3826. 19189669

[B136] Tunggal J. A. Helfrich I. Schmitz A. Schwarz H. Günzel D. Fromm M. (2005). E-cadherin is essential for *in vivo* epidermal barrier function by regulating tight junctions. EMBO J. 24 (6), 1146–1156. 10.1038/sj.emboj.7600605 15775979 PMC556407

[B137] Ungewiß H. Vielmuth F. Suzuki S. T. Maiser A. Harz H. Leonhardt H. (2017). Desmoglein 2 regulates the intestinal epithelial barrier via p38 mitogen-activated protein kinase. Sci. Rep. 7 (1), 6329–6410. 10.1038/s41598-017-06713-y 28740231 PMC5524837

[B138] Valenta T. Hausmann G. Basler K. (2012). The many faces and functions of β-catenin. Eur. Mol. Biol. Organ 31 (12), 2714–2736. 10.1038/emboj.2012.150 PMC3380220 22617422

[B139] van Hengel J. van Roy F. (2007). Diverse functions of p120ctn in tumors. Biochimica Biophysica Acta (BBA) - Mol. Cell Res. 1773 (1), 78–88. 10.1016/j.bbamcr.2006.08.033 17030444

[B140] van Hengel J. Vanhoenacker P. Staes K. van Roy F. (1999). Nuclear localization of the p120 ctn Armadillo-like catenin is counteracted by a nuclear export signal and by E-cadherin expression. Proc. Natl. Acad. Sci. U.S.A. 96 (14), 7980–7985. 10.1073/pnas.96.14.7980 10393933 PMC22173

[B141] Van Itallie C. M. Tietgens A. J. Aponte A. Fredriksson K. Fanning A. S. Gucek M. (2014). Biotin ligase tagging identifies proteins proximal to E-cadherin, including lipoma preferred partner, a regulator of epithelial cell-cell and cell-substrate adhesion. J. Cell Sci. 127 (4), 885–895. 10.1242/jcs.140475 24338363 PMC3924204

[B142] Van Patten K. Parkash V. Jain D. (2010). Cadherin expression in gastrointestinal tract endometriosis: Possible role in deep tissue invasion and development of malignancy. Mod. Pathol. 23 (1), 38–44. 10.1038/modpathol.2009.127 19898423

[B143] Vivinus-Nébot M. Frin-Mathy G. Bzioueche H. Dainese R. Bernard G. Anty R. (2014). Functional bowel symptoms in quiescent inflammatory bowel diseases: Role of epithelial barrier disruption and low-grade inflammation. Gut 63 (5), 744–752. 10.1136/gutjnl-2012-304066 23878165

[B144] Weber A. Marquardt J. Elzi D. Forster N. Starke S. Glaum A. (2008). Zbtb4 represses transcription of P21CIP1 and controls the cellular response to p53 activation. EMBO J. 27 (11), 1563–1574. 10.1038/emboj.2008.85 18451802 PMC2426723

[B145] White B. D. Chien A. J. Dawson D. W. (2012). Dysregulation of wnt/β-catenin signaling in gastrointestinal cancers. Gastroenterology 142 (2), 219–232. 10.1053/j.gastro.2011.12.001 22155636 PMC3285553

[B146] Wong N. A. C. S. Pignatelli M. (2002). β-Catenin-A linchpin in colorectal carcinogenesis? Am. J. Pathology 160 (2), 389–401. 10.1016/s0002-9440(10)64856-0 PMC1850660 11839557

[B147] Xiang T. He K. Wang S. Chen W. Li H. (2020). Expression of zinc finger and BTB domain-containing 4 in colorectal cancer and its clinical significance. Cmar 12, 9621–9626. 10.2147/cmar.s266529 PMC7547138 33116821

[B148] Xiao K. Oas R. G. Chiasson C. M. Kowalczyk A. P. (2007). Role of p120-catenin in cadherin trafficking. Biochimica Biophysica Acta (BBA) - Mol. Cell Res. 1773 (1), 8–16. 10.1016/j.bbamcr.2006.07.005 16949165

[B149] Xiong Y. Kotake Y. (2006). No exit strategy? No problem: APC inhibits β-catenin inside the nucleus: Figure 1. Genes Dev. 20 (6), 637–642. 10.1101/gad.1413206 16543216

[B150] Yamada S. Pokutta S. Drees F. Weis W. I. Nelson W. J. (2005). Deconstructing the cadherin-catenin-Actin complex. Cell 123 (5), 889–901. 10.1016/j.cell.2005.09.020 16325582 PMC3368712

[B151] Yang J. Zhang W. Evans P. M. Chen X. He X. Liu C. (2006). Adenomatous polyposis coli (APC) differentially regulates β-catenin phosphorylation and ubiquitination in colon cancer cells. J. Biol. Chem. 281 (26), 17751–17757. 10.1074/jbc.m600831200 16798748

[B157] Yap A. S. Niessen C. M. Gumbiner B. M. (1998). The juxtamembrane region of the cadherin cytoplasmic tail supports lateral clustering, adhesive strengthening, and interaction with p120(ctn). J. Cell Biol. 141 (3), 779–789. 9566976 10.1083/jcb.141.3.779 PMC2132752

[B152] Yokoya F. Imamoto N. Tachibana T. Yoneda Y. (1999). β-Catenin can Be transported into the nucleus in a ran-unassisted manner. MBoC 10 (4), 1119–1131. 10.1091/mbc.10.4.1119 10198061 PMC25239

[B153] Yu Y. Shang R. Chen Y. Li J. Liang Z. Hu J. (2018). Tumor suppressive ZBTB4 inhibits cell growth by regulating cell cycle progression and apoptosis in Ewing sarcoma. Biomed. Pharmacother. 100, 108–115. 10.1016/j.biopha.2018.01.132 29425745

[B154] Zhang S. Wang Z. Shan J. Yu X. Li L. Lei R. (2016). Nuclear expression and/or reduced membranous expression of β-catenin correlate with poor prognosis in colorectal carcinoma. Med. (United States). 95 (49), e5546. 10.1097/md.0000000000005546 PMC5266024 27930552

[B155] Zhao Y. Yu T. Zhang N. Chen J. Zhang P. Li S. (2019). Nuclear E-cadherin acetylation promotes colorectal tumorigenesis via enhancing β-catenin activity. Mol. Cancer Res. 17 (2), 655–665. 10.1158/1541-7786.mcr-18-0637 30401720

[B156] Zhurinsky J. Shtutman M. Ben-Ze'ev A. (2000). Plakoglobin and beta-catenin: Protein interactions, regulation and biological roles. J. Cell Sci. 113 (18), 3127–3139. 10.1242/jcs.113.18.3127 10954412

